# Whole‐mitogenome analysis unveils previously undescribed genetic diversity in cane toads across their invasion trajectory

**DOI:** 10.1002/ece3.11115

**Published:** 2024-03-03

**Authors:** Kelton Cheung, Timothy G. Amos, Rick Shine, Jayna L. DeVore, Simon Ducatez, Richard J. Edwards, Lee Ann Rollins

**Affiliations:** ^1^ Evolution & Ecology Research Centre, School of Biological, Earth & Environmental Sciences University of New South Wales Sydney New South Wales Australia; ^2^ School of Biotechnology & Biomolecular Sciences University of New South Wales Sydney New South Wales Australia; ^3^ Garvan Institute of Medical Research Sydney New South Wales Australia; ^4^ Department of Biological Sciences Macquarie University Sydney New South Wales Australia; ^5^ Univ. Polynésie Francaise UMR 241 EIO (UPF, IRD, IFREMER, ILM) BP 6570 Faa'a Tahiti French Polynesia; ^6^ Institut de Recherche pour le Développement (IRD) UMR 241 EIO (UPF, IRD, IFREMER, ILM) BP 6570 Faa'a Tahiti French Polynesia; ^7^ Minderoo OceanOmics Centre at UWA, Oceans Institute The University of Western Australia Perth Western Australia Australia

**Keywords:** haplotypes, invasion biology, mitochondrial genome, population genomics, *Rhinella marina*

## Abstract

Invasive species offer insights into rapid adaptation to novel environments. The iconic cane toad (*Rhinella marina*) is an excellent model for studying rapid adaptation during invasion. Previous research using the mitochondrial NADH dehydrogenase 3 (*ND3*) gene in Hawai'ian and Australian invasive populations found a single haplotype, indicating an extreme genetic bottleneck following introduction. Nuclear genetic diversity also exhibited reductions across the genome in these two populations. Here, we investigated the mitochondrial genomics of cane toads across this invasion trajectory. We created the first reference mitochondrial genome for this species using long‐read sequence data. We combined whole‐genome resequencing data of 15 toads with published transcriptomic data of 125 individuals to construct nearly complete mitochondrial genomes from the native (French Guiana) and introduced (Hawai'i and Australia) ranges for population genomic analyses. In agreement with previous investigations of these populations, we identified genetic bottlenecks in both Hawai'ian and Australian introduced populations, alongside evidence of population expansion in the invasive ranges. Although mitochondrial genetic diversity in introduced populations was reduced, our results revealed that it had been underestimated: we identified 45 mitochondrial haplotypes in Hawai'ian and Australian samples, none of which were found in the native range. Additionally, we identified two distinct groups of haplotypes from the native range, separated by a minimum of 110 base pairs (0.6%). These findings enhance our understanding of how invasion has shaped the genetic landscape of this species.

## Graphical abstract

The iconic cane toad (*Rhinella marina*) serves as an excellent model for studying rapid adaptation during invasion. We created the first reference mitochondrial genome for this species using long‐read sequence data. We combined whole‐genome resequencing data of 15 toads with published transcriptomic data of 125 individuals to construct nearly complete mitochondrial genomes from the native (French Guiana) and introduced (Hawai'i and Australia) ranges for population genomic analyses. We revealed genetic bottlenecks post‐introduction and an underestimation of genetic diversity in the introduced populations.

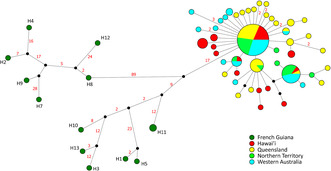

## 1 INTRODUCTION

Invasive species are a substantial global concern, particularly due to likely future range expansions under climate change (Bonebrake et al., 2018; Seebens et al., 2017), an increasing rate of anthropogenic introductions (Lowe et al., 2000), and their negative impacts on native biodiversity and the economy (Bradshaw et al., 2016; Mainka & Howard, 2010). Biological invasions sometimes present a genetic paradox where, despite small founding population sizes that often reduce genetic diversity in the introduced range (i.e. genetic bottlenecks), they still maintain the ability to readily adapt to novel conditions (Allendorf & Lundquist, 2003; Estoup et al., 2016; Schrieber & Lachmuth, 2017). Many invasive populations have shown evidence of adaptation to their new environments (Rollins et al., 2013), creating an opportunity to study the process of rapid evolution.

Various genetic markers, including those from the nuclear and mitochondrial genomes, have been employed to investigate the introduction history and evolutionary processes of invasive species (McGaughran et al., 2024). Because of their distinct mode of inheritance and mutation rates, nuclear and mitochondrial markers may yield different insights into the evolutionary events underlying invasion (Toews & Brelsford, 2012). Genetic variation in invasive species is shaped by complex interplays among genetic drift, demography, and selection. Small founding populations, common during an invasion, can promote genetic drift, leading to reduced genetic diversity. Furthermore, while genetic drift results in less efficient selection (Gravel, 2016), populations expanding their range into new environments may experience novel selection regimes (Sakai et al., 2001) and spatial sorting (Shine et al., 2011) that further reduce genetic diversity.

Mitochondrial DNA (mtDNA) is more susceptible to genetic drift than nuclear DNA due to the former's uniparental inheritance and smaller effective population size (Klucnika & Ma, 2019; Xiao et al., 2017). Nonetheless, mtDNA has been extensively used to study the evolutionary history of different species or populations within the same species because of its non‐recombining nature and purported near‐neutrality. However, the neutrality of mtDNA has been challenged by evidence of selection, likely due to the important role of mitochondria in ATP production and cellular activities (Meiklejohn et al., 2007). Deleterious mutations are rapidly removed by purifying selection to maintain the function of the electron transport chain. Furthermore, variation in mtDNA has been linked to adaptation to high latitudes (Chen et al., 2020; Wang et al., 2021) and temperature (Baker et al., 2019; Cheng et al., 2013; Silva et al., 2014). Despite these challenges, there exists a substantial repository of mtDNA data in public databases, enabling comprehensive comparative genetic analyses, increasing its usefulness as a marker.

The cane toad (*Rhinella marina*) is one of the world's most infamous, and well‐studied, invasive species (Lowe et al., 2000). Cane toads are native to Central and South America (Zug & Zug, 1979) and have been introduced to multiple Caribbean islands for biological control of cane beetles, a parasite of sugarcane (Easteal, 1981). Cane toads were transported from the Caribbean to Hawai'i in 1932 for cane beetle control, and in 1935, 101 Hawai'ian cane toads were translocated from Oahu to Queensland, Australia (Turvey, 2013). However, they failed to control cane beetles in Australia and, facilitated by a high reproductive rate, subsequently spread across the continent to arid Western Australia. This species' inexorable spread throughout its introduced range in Australia has had catastrophic effects on the naïve predators it has encountered (Chinchio et al., 2020). Freshwater crocodiles, snakes, lizards, and quolls have been particularly badly affected by the cane toad invasion in Australia (Shine, 2010). The colonisation of the cane toad is still ongoing; toads occupy over 1.2 million km^2^ of Australia (Urban et al., 2008) including tropical Queensland, the Northern Territory, and the Kimberley region of northern Western Australia (Radford et al., 2019).

Like in many other invasions, the cane toads' translocation resulted in genetic bottlenecks. Australian cane toads have very low major histocompatibility complex (MHC) class I and class II (Lillie et al., 2014, 2016, 2017) and microsatellite (Leblois et al., 2000) diversity. Previous analysis of the NADH dehydrogenase 3 (*ND3*) mitochondrial gene from 31 individuals sampled from Hawai'i and Australia identified a single haplotype, a substantial reduction of haplotype diversity compared to the five haplotypes observed in the native range (Slade & Moritz, 1998), further supporting the presence of genetic bottlenecks in the invasive ranges. Despite this low genetic variation, toads from the Australian range differ substantially in immune function, dispersal ability, and behaviour (Alford et al., 2009; Brown, Phillips, Dubey, & Shine, 2015; Gruber et al., 2018; Gruber, Whiting, Brown, & Shine, 2017; Phillips et al., 2010). Some of these traits have been shown to be intergenerationally transmitted, consistent with genetic causes. For example, toads from range‐core and range‐edge populations that were raised in a common‐garden experiment showed divergence of behavioural traits and morphology (Gruber, Brown, Whiting, & Shine, 2017; Hudson, Brown, & Shine, 2016). The invasion success of this species in Australia, coupled with evidence of evolutionary change since introduction, makes it an ideal system for the study of rapid evolution during invasion.

Despite extensive research into toad evolution in the introduced range, we need improved genetic resources to clarify the molecular mechanisms underlying these changes. Transcriptome data from spleen, muscle, and/or brain tissues of toads from French Guiana, Hawai'i, and Australia and liver tissue from Brazil exist (Richardson et al., 2018; Rollins et al., 2015; Selechnik, Richardson, Shine, Brown, & Rollins, 2019; Yagound et al., 2022), and a draft reference genome (Edwards et al., 2018) is available, but population genomic analyses of cane toads across this invasion trajectory have thus far been restricted to a single study of single‐nucleotide polymorphisms (SNPs) from the nuclear genome (Selechnik, Richardson, Shine, DeVore, et al., 2019). Here, we present the first complete cane toad mitochondrial genome (mitogenome), assembled from the same Western Australian individual as the draft nuclear genome (Edwards et al., 2018). We then investigate evolution across the invasion trajectory using mtDNA data derived from RNA‐Seq and whole‐genome resequencing (WGS). We compare a subset of our data to those of a previously published study of *ND3* on the same populations (Slade & Moritz, 1998), to determine whether our whole‐mitochondrial genome analysis can extend population genetic inferences. We predicted that there would be decreasing genetic diversity within the mitochondrial genome with increasing distance from the native range due to genetic bottlenecks. We also predicted that we would identify more genetic diversity within Australia than has been previously characterised, given the phenotypic evidence of adaptation to this novel environment.

## 2 MATERIALS AND METHODS

### 2.1 Reference mitochondrial genome

High‐molecular‐weight genomic DNA was extracted from the liver of a female cane toad collected in the Kimberley region of Western Australia and sequenced on the Illumina HiSeq X Ten and PacBio RS II platforms as described previously (Edwards et al., 2018). We assembled the mitogenome from 36 SMRT cells generated from the first two SMRTBell libraries from Edwards et al. (2018) (ENA experiments ERX2389178 and ERX2389179).

We downloaded the 17,757 base pair (bp) *Bufo japonicus* complete mitochondrial genome (gi: 157939553) from NCBI on 29 March 2016 and used it to identify mitochondrial subreads from the raw sequencing data. We used GABLAM (Davey et al., 2006) to map the *B. japonicus* mtDNA onto cane toad PacBio subreads using blastn (blast+ v2.2.31) and extracted 143 subreads that had 75% + bidirectional coverage (e.g. at least 75% of the subread mapped onto the mtDNA and at least 75% of the mtDNA was covered by the subread). We reverse complemented any subreads that mapped the negative strand. We identified the subread with the best global hit to the *B. japonicus* mtDNA and performed a second GABLAM search of all 143 subreads against this new query, retaining 29 sequences that had >80% global sequence identity within local matches in the same orientation and order, excluding any “wrapping” due to circularity. We aligned these 29 sequences using MAFFT v2.273 (Katoh & Standley, 2013) with a very small gap penalty to account for the high indel rate (‐‐localpair ‐‐maxiterate 1000 ‐‐op 0.1 ‐‐ep 0.1 ‐‐lop ‐0.1 ‐‐nuc). Using SeqSuite (Edwards et al., 2020), we generated a consensus sequence from the alignment using the most frequent non‐gap base call at each position. We excluded from the consensus sequence any column with fewer than 5 non‐gap sequences. We mapped all 143 raw subreads onto the consensus sequence using BLASR and polished it using Quiver (SMRT Analysis v2.3.0). We used GABLAM to identify the overlapping ends of the sequence, which was then circularised by trimming off the first 1957 bp for a final length of 18,152 bp. We performed a final polishing step using the Illumina data (ENA experiment ERX2845325), mapped with Bowtie v2.3.3 (Langmead & Salzberg, 2012) and error‐corrected with Pilon v1.20 (Walker et al., 2014).

We annotated the mitochondrial genome using MITOS online (Bernt et al., 2013) with the vertebrate mtDNA genetic code (Genetic Code Table 02). We manually curated the annotation of protein‐coding genes and associated stop codons where required: where no canonical in‐frame stop codon was found, we assumed that a partial (T or TA) stop codon would be completed upon polyadenylation. We compared the gene arrangement of the mitochondrial genome to the closely related *Rhinella* species (*Rhinella* cf. *acrolopha*) (accession number: KT221613) (Machado et al., 2016). We calculated codon usage using https://www.bioinformatics.org/sms2/codon_usage.html (accessed on 29 March 2023). We uploaded the final annotated mitochondrial genome to NCBI (accession number: NC_066225.1). We drew the mitogenome map using OGDRAW (Greiner et al., 2019).

### 2.2 Sequence data for population‐ and species‐level analyses

**FIGURE 1:**
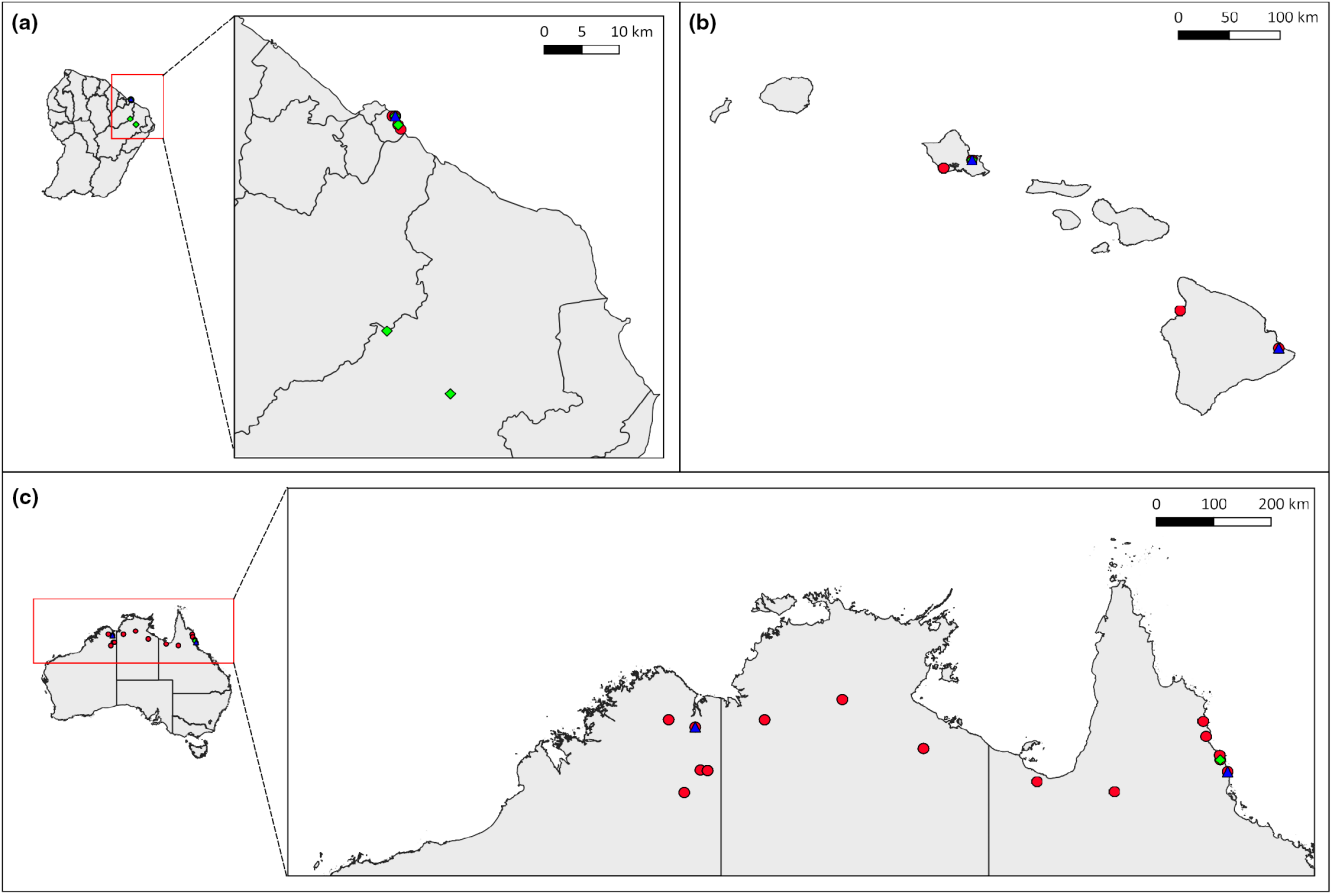
Sample collection sites of the cane toad (*Rhinella marina*) in (a) French Guiana (the native range), (b) Hawai'i (invasive range), and (c) Australia (invasive range) for RNA‐Seq and DNA‐Seq. Red dots indicate samples for RNA‐Seq, green squares indicate samples for DNA‐Seq, and blue triangles indicate samples with both RNA‐Seq and DNA‐Seq.

We downloaded previously published cane toad RNA‐Seq data from different tissues from NCBI BioProject PRJNA510261 and PRJNA395127 (spleen), PRJNA479937 (brain), and PRJNA277985 (muscle) for analysis. We included a total of 125 individuals comprising 144 tissue samples, from individuals sampled in French Guiana (*n* = 24), Hawai'i (*n* = 8), and Australia (*n* = 93) (Figure 1; Table S1). Where multiple tissues were available for an individual (brain and spleen), we used the more common tissue type (brain). We processed raw reads as described previously (Selechnik, Richardson, Shine, DeVore, et al., 2019) with STAR v2.7.2b (Dobin et al., 2013) and Genome Analysis Toolkit (GATK) v4.1.9.0 (McKenna et al., 2010), using the extracted mitogenome from the draft genome as a reference.

We produced WGS data for an additional 15 cane toad samples (see “WGS” in Table S1) to provide a broader geographical context in the native range and increase our whole‐mitochondrial genome population genetic analysis sample size, bringing our total number of individuals included to 132. Individuals were chosen where possible to enable comparison of DNA‐Seq‐ and RNA‐Seq‐derived mitogenomes (8 individuals). We extracted genomic DNA using a Gentra PureGene DNA extraction kit (QIAGEN) (Figure 1, Table S1). For two of the samples (RMH006 and RM265), paired‐end sequencing libraries were prepared using the TruSeq DNA PCR‐Free kit (Illumina) and then sequenced on the Illumina NovaSeq 6000 platform at the Ramaciotti Centre for Genomics, University of New South Wales, Sydney, Australia (UNSW Sydney). For the other 13 samples, paired‐end sequencing libraries were prepared according to the manufacturer's specifications for the NEBNext Ultra DNA Library Prep Kit for Illumina (New England Biolabs). Three of these (samples RMF031, RMF042, and RMF044) were then sequenced on the 10X Genomics Chromium platform at the Ramaciotti Centre for Genomics, UNSW Sydney. The remaining 10 libraries were sequenced on the Illumina NovaSeq 6000 platform at Deakin Genomics Centre, Victoria, Australia, with a target depth of 20‐fold coverage per genome. We processed raw sequencing reads as described above.

To put our data into the context of previous studies (Acevedo et al., 2016; Slade & Moritz, 1998), we obtained the mitochondrial *ND3* gene with tRNA‐Gly and tRNA‐Arg flanking sequence (*ND3* dataset hereafter) data from NCBI (Table S1). We aligned our data with this *ND3* dataset using the R package DECIPHER (Wright, 2016) and trimmed our data to match the pre‐existing sequences.

### 2.3 Construction of mtDNA genome and variant calling

We used GATK HaplotypeCaller (Poplin et al., 2018) to call SNPs and indels using the following criteria: soft clipped bases were not included, and variants with minimum Phred‐scaled confidence of 20 threshold were kept. To filter low‐quality variants, we used GATK VariantFiltration (Van der Auwera, 2020) to remove variants in the resulting VCF file: clusters of 3 SNPs within a window of 35 bases, variants with FisherStrand (FS) >30.0, QualByDepth (QD) <2.0, depth of coverage (DP) <20.0, and allelic frequency (AF) <0.05. Due to the difficulty of mapping short‐read RNA‐Seq data to sequences with multiple repeats and the risk of introducing false‐positive variant calls, we excluded variants from the repetitive sequences in the hypervariable region 2 (HV2) of the mitochondrial control region. We determined the consensus sequence of each sample using FastaAlternateReferenceMaker (McKenna et al., 2010).

We aligned consensus mitogenome sequences from individuals with both RNA‐Seq and WGS data against each other using DECIPHER R packages (Wright, 2016, 2020) using default settings. We further visualised and analysed the alignments with AliView (Larsson, 2014).

### 2.4 Nuclear mitochondrial DNA segment (NUMT) identification

The presence of NUMTs in the cane toad's genome has not been documented previously. Because NUMTs can inflate estimates of genetic variations (Song et al., 2008), it is prudent to investigate their presence in mitogenome population genetic studies. We downloaded the cane toad draft genome from NCBI BioProject PRJEB24695. We used RepeatModeler (http://www.repeatmasker.org/RepeatModeler/) to construct species‐specific repeat library and masked the genome using RepeatMasker (Tarailo‐Graovac & Chen, 2009). We used NUMTFinder v0.5.3 (Edwards et al., 2021) to identify potential NUMTs by searching the full and repeat‐masked versions of the nuclear genome with our new mtDNA assembly.

### 2.5 Population genetic analyses

We examined the spatial distribution of haplotypes by creating a median‐joining haplotype network using Network and postprocessed using the maximum parsimony calculation to remove unnecessary median vectors and links (Polzin & Vahdati Daneshmand, 2003). We drew the final network using Network Publisher v2.1.2.5 (Fluxus Engineering, Clare, UK). This same procedure also was used to analyse the *ND3* dataset (Table S1; Slade & Moritz, 1998).

We then investigated population genetic diversity and differentiation. We calculated the following diversity indices using DnaSP v6 (Rozas et al., 2017): nucleotide diversity (*π*), number of polymorphic sites, number of parsimony‐informative sites, number of haplotypes (*H*), and haplotype diversity (*h*). Due to the uneven sample size across populations, we calculated haplotype richness (*H*_R_) using FSTAT v2.9.4 (Goudet, 1995) to measure the number of alleles independent of the sample size. We used DnaSP to generate a haplotype list for Arlequin software v3.5.2.2 (Excoffier & Lischer, 2010) and a Roehl data file for use with Network software 10.2.0.0 (Bandelt et al., 1995, 1999). To study the population structure, we employed Arlequin to calculate genetic differentiation among and between populations using pairwise fixation indices (*F*_st_) and analysis of molecular variance (AMOVA) to estimate the genetic variation among and within populations.

To study the demographic history of each population, we used both neutrality statistics and mismatch distribution tests to infer population expansion. We produced Fu's *F*s statistics implemented in Arlequin and estimated Ramos‐Onsins and Rozas *R* ^2^ statistics using DnaSP. Fu's *F*s test calculates the probability of observing a number of haplotypes similar to or smaller than the observed number of samples (Fu, 1997). Both tests are based on the infinite‐site model without recombination, which is suitable for analysing mitochondrial genomic data. A significant negative value of *F*s indicates an excess of rare alleles and haplotypes due to a sudden population growth after a population bottleneck or genetic hitchhiking, while positive values indicate a lack of rare mutations, resulting from balancing selection or population stability. Ramos‐Onsins and Rozas *R* ^2^ statistic compares the difference between the number of singleton mutations and the average number of segregating sites (Ramos‐Onsins & Rozas, 2002). The *R* ^2^ statistic is known to have better power than Fu's *F*s when the sample size is small (<10) or the number of segregating sites is low (Ramirez‐Soriano et al., 2008). A small positive value of *R* ^2^ indicates population expansion. We calculated the statistical significance of these tests using 10,000 simulations. We constructed mismatch distributions of the frequency of pairwise differences between haplotype pairs in each population using the sudden expansion and spatial expansion models implemented in Arlequin (Excoffier, 2004; Schneider & Excoffier, 1999). We used the sum of squared deviations (SSD) to identify differences between observed and expected values and used the raggedness index to explore the smoothness of the observed mismatch distribution. We calculated the statistical significance of these tests using 500 simulated replicates in Arlequin. A non‐significant SSD value and a small raggedness index value with a unimodal distribution indicate no deviation of the observed data from the expectation under the expansion model.

## 3 RESULTS

### 3.1 Summary of the cane toad reference mitochondrial genome

**FIGURE 2:**
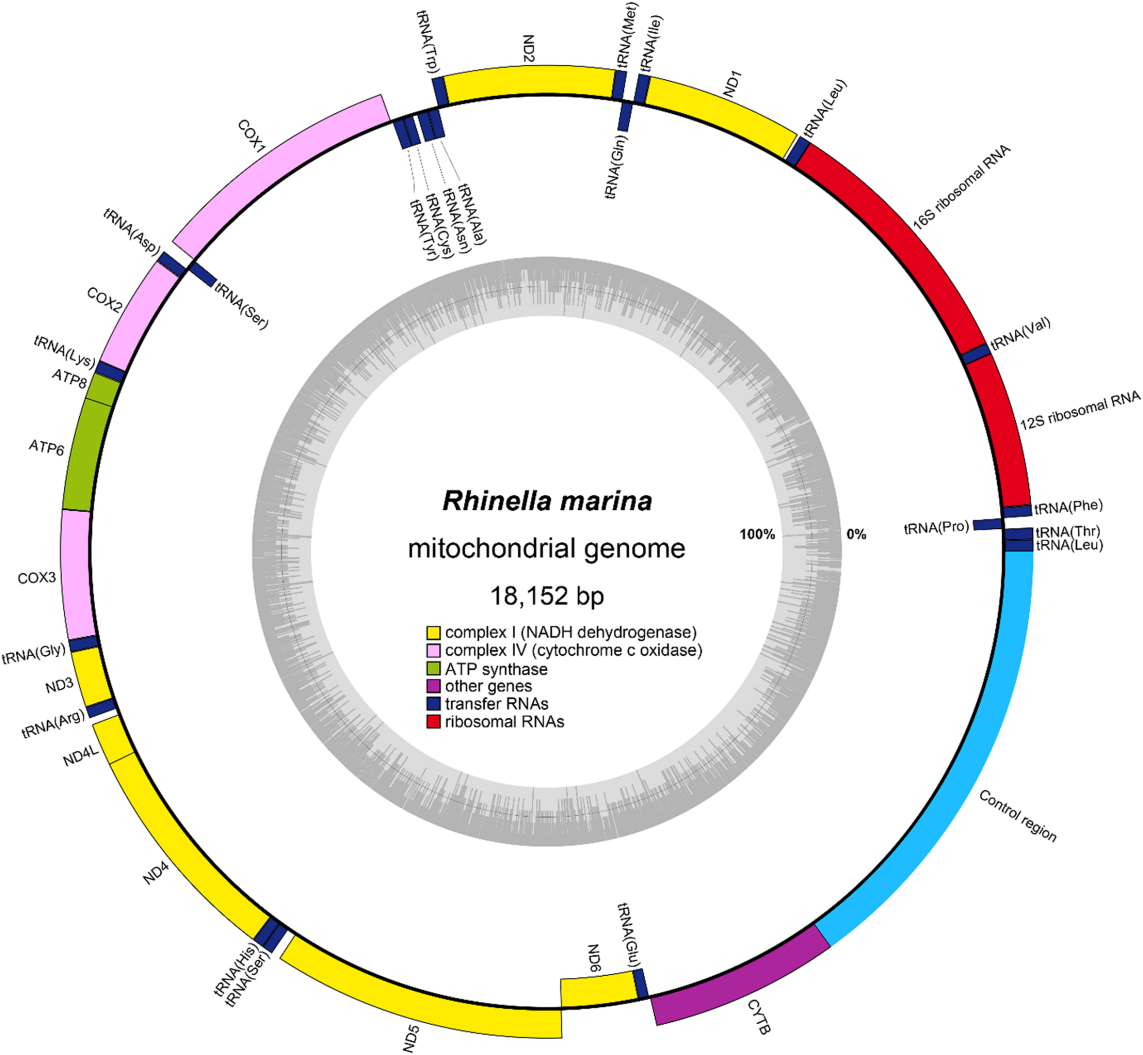
Mitochondrial genome structure of *Rhinella marina*. Genes are coloured according to the functional categories shown in the legend. Genes on the positive strand are on the outside of the outer circle, and genes on the negative strand are on the inside of the outer circle. The inner grey graph shows the GC content of the mitogenome. The starting and stop positions of each gene are described in Table S2.

The mitogenome of *R. marina* (Figure 2) consists of 18,152 bp, including 22 tRNA genes, 2 rRNA genes, and 13 protein‐coding genes, with 28 genes located on the forward (+) strand and 9 located on the reverse (−) strand (Table S2). There is also a 2753 bp GC‐rich control region containing the origin of replication. Two groups of tandem repeats were observed in the latter part of the control region: 530 bps with 6‐full repeats and 350 bps with 3‐full repeats. The gene order of the cane toad mitogenome was examined by comparing it to the closely related *Rhinella* cf. *acrolopha*, and we found no gene rearrangements (data not shown). The base composition of the mitogenome was A: 29%, T: 33%, G: 14%, and C: 24%, with a bias with 62% of A + T. The codon usages of those with nucleotide G in the third codon position were lower than those with A and T. GCG (Ala) was the least used codon, and TGG was the only codon used for encoding tryptophan. All protein‐coding genes used ATG as the start codon.

### 3.2 Verification of mtDNA genomes from RNA‐Seq data

A near‐complete mitochondrial genome with 17,261 to 17,270 bps (95.1% of reference mitochondrial genome) for a total of 132 individuals was generated from the RNA‐Seq and WGS dataset. A sequence with length of 889 bases consisting of repeats in the HV2 section of the control region was excluded due to difficulty in correctly mapping sequencing reads on repeat regions. To determine the accuracy of our RNA‐Seq‐derived mtDNA sequences, we then compared the sequences of the eight individuals for which we had both RNA‐Seq and WGS data (Table S3). We identified four sites (site 2244, site 5162, site 7162, and site 7920) that showed intra‐individual variation across the two datasets in multiple individuals (i.e. potential RNA editing). Two sites in coding regions (site 267 and site 477) had intra‐individual differences in only one individual. Additionally, one individual had 12 sites in the control region that differed across datasets (Table S3). We replaced the sites with intra‐individual variation in multiple individuals with Ns, minimising variation for subsequent population analyses that might be introduced by RNA editing.

### 3.3 NUMT analysis

We found 42 short mtDNA fragments (38–140 bps in length) that mapped to 42 individual contigs of the cane toad draft nuclear genome with over 80% identity (Table S4). All fragments corresponded to the control region of the mitogenome. After the draft genome was masked for repeat elements, no mtDNA fragments were found to map to the nuclear genome.

### 3.4 Population genetic analyses

**FIGURE 3:**
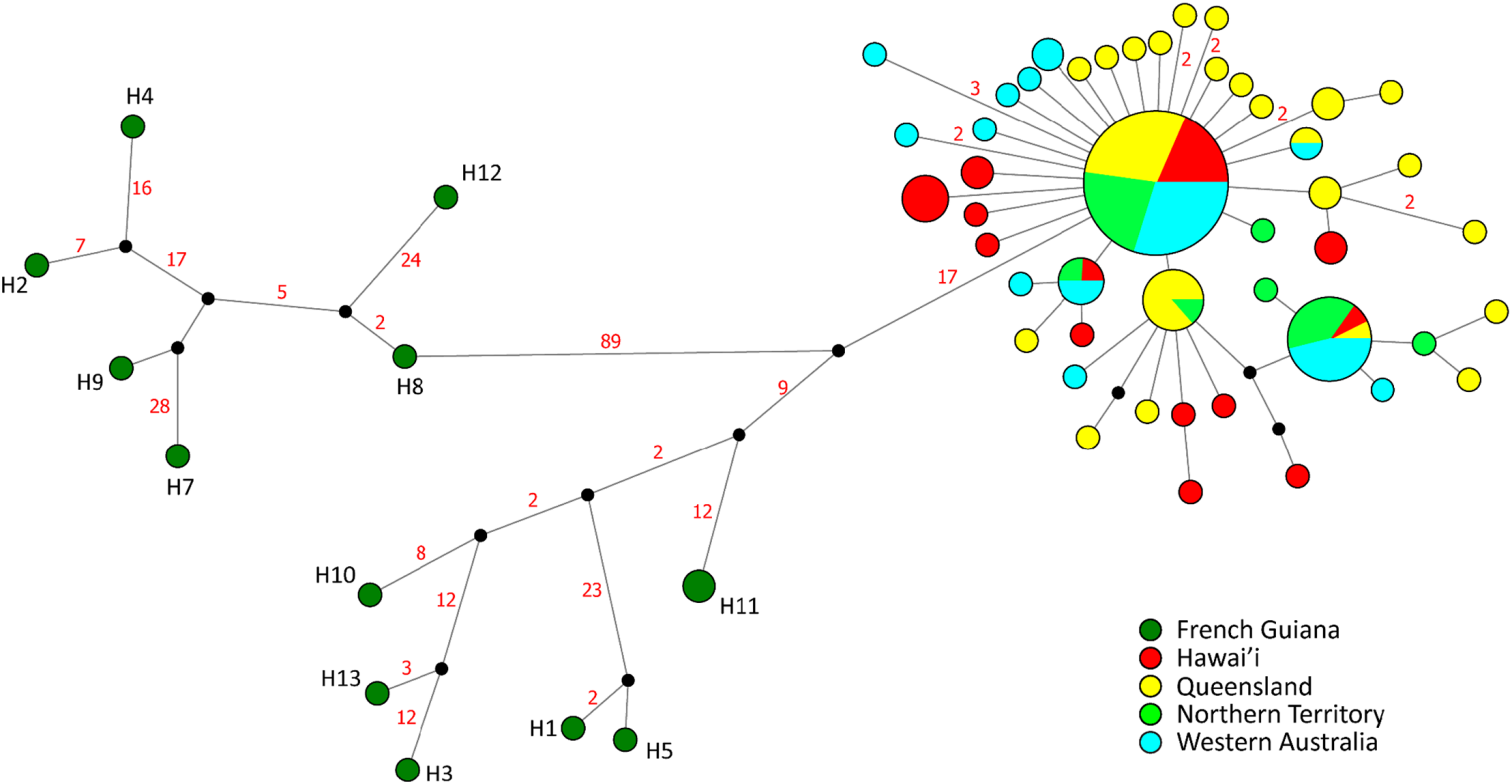
The haplotype network illustrating the relationships among 132 cane toads (*Rhinella marina*) from French Guinea, Hawai'i, and Australia based on near‐complete mitochondrial genomes. Each sample location is represented by a different colour. A black solid circle represents a putative haplotype that was not directly sampled. The red number adjacent to a line indicates the number of nucleotide substitutions between haplotypes, while the absence of a number indicates a single‐nucleotide substitution.

We identified 262 (1.30%) polymorphic sites in the RNA‐Seq dataset, 198 of which were parsimony‐informative, including 25 from RNA genes, 145 from protein‐coding genes, and 28 from non‐coding regions and the control region (Table S2), which resulted in the identification of 58 haplotypes (Table 1; Figure 3). The 13 native samples contained 12 unique haplotypes. Two haplotypes found in all introduced populations accounted for nearly 50% of the sampled individuals. Within introduced populations, a “complex‐star” shape topology with mostly singleton mutants was observed, and 41 of the 46 introduced haplotypes (89%) were private to a single introduction (Hawai'i: 10; Australia: 31).

**TABLE 1:** Population genetic statistics of native‐range population (French Guiana) and introduced populations (Hawai'i and Australia).

	*N*	Polymorphic sites	*H*	*π* ± SD	*h* ± SD	*H*_R_
French Guiana	13	205	12	0.00469 ± 0.00037	0.987 ± 0.035	12
Hawai'i	25	11	13	0.00010 ± 0.00002	0.880 ± 0.051	10.7
Australia	94	40	36	0.00009 ± 0.00001	0.834 ± 0.035	12.5
Queensland	42	27	23	0.00012 ± 0.00001	0.890 ± 0.040	13.5
Northern Territory	20	5	7	0.00005 ± 0.00001	0.711 ± 0.089	6.4
Western Australia	32	16	13	0.00008 ± 0.00001	0.809 ± 0.06	9.6

Abbreviations: *h*, haplotype diversity; *H*, number of haplotypes; *H*_R_; haplotype richness; *N*, number of samples; *π*, nucleotide diversity.

**FIGURE 4:**
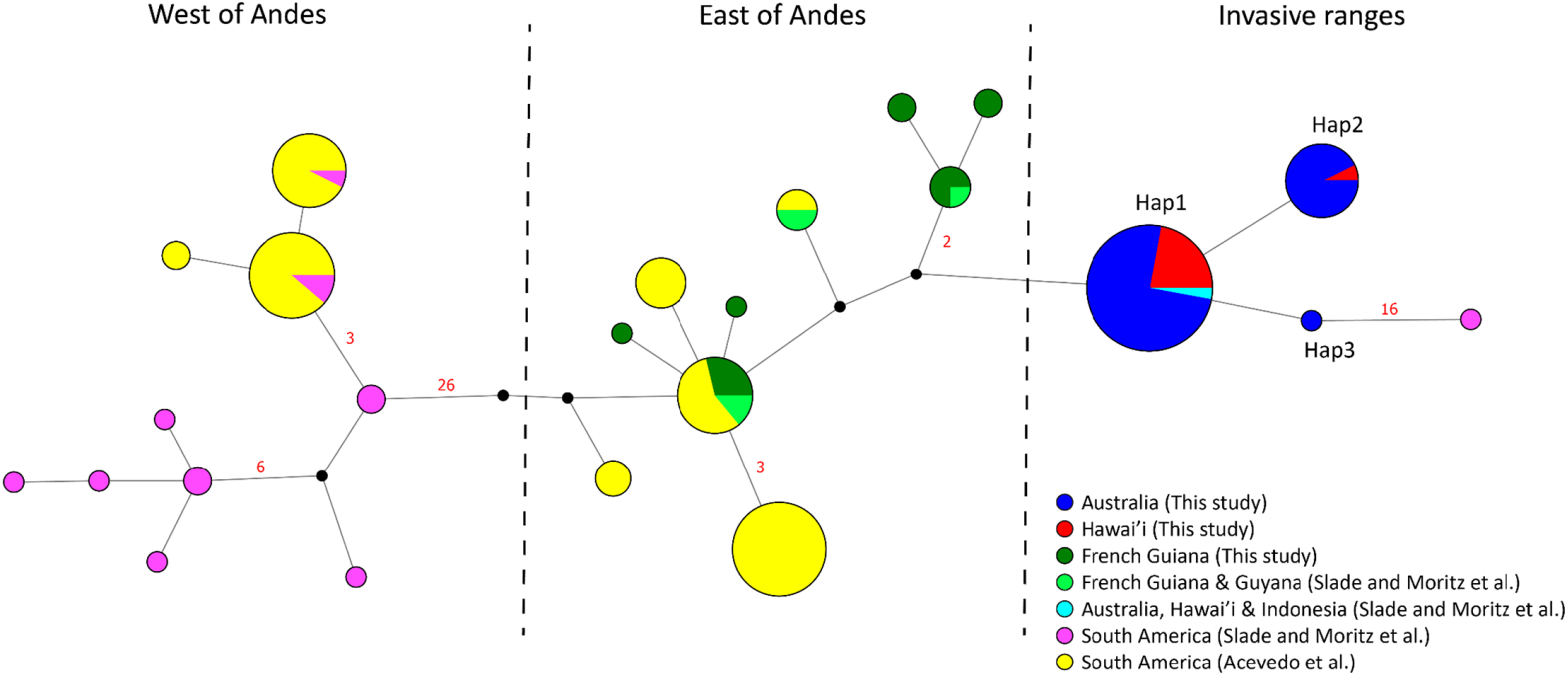
Haplotype network of cane toads (*Rhinella marina*) from native and invasive ranges. Network built using a 510 bp segment of ND3 with flanking tRNAs. Included are a total of 223 samples, incorporating samples from this study, Slade and Moritz (1998), and Acevedo et al. (2016). Sample locations for each study are represented by different colours. A black solid circle represents a putative haplotype that was not directly sampled. The red number adjacent to a line indicates the number of nucleotide substitutions between haplotypes, while the absence of a number indicates a single‐nucleotide substitution. Native‐range samples are separated by the Andes Mountains in South America (Acevedo et al., 2016). Toads on the west side of the Andes are now known as *Rhinella horribilis*. Invasive samples are most similar to cane toads from the east of the Andes.

We also compared the nearly complete mitochondrial genome dataset with the *ND3* dataset to demonstrate the consistency of haplotype inference across studies and to put our data into the context of previous work. A total of 24 haplotypes were identified in this analysis (Figure 4): six haplotypes were identified in French Guiana/Guyana, one haplotype was identified in Indonesian samples, three in Hawai'ian and Australian samples, and the remaining haplotypes were from other South American localities. Haplotypes from either side of the Andes clearly formed distinct lineages, which is consistent with previous work (Acevedo et al., 2016). The single Australian haplotype (Hap1) previously identified in (Slade & Moritz, 1998) was the most common haplotype in our Australian samples, and the other two haplotypes (Hap2 and Hap3) within Australia were a single nucleotide different to this common haplotype.

**FIGURE 5:**
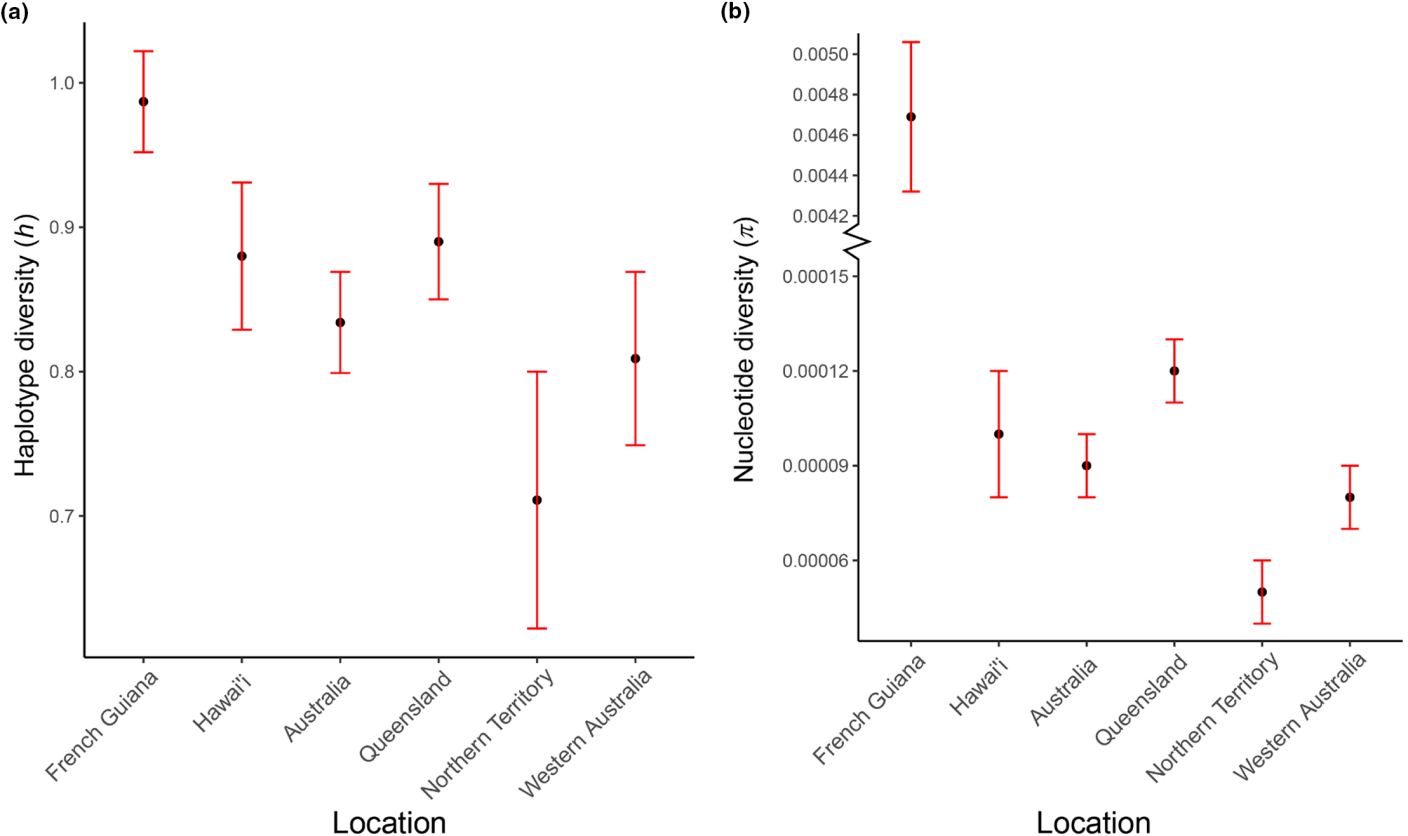
Haplotype diversity (a) and nucleotide diversities (b) of native population and introduced populations.

Haplotype diversity was highest in the native range, with reduced diversity in both Hawai'ian and Australian populations (*h*: 0.9782 vs. 0.8800 and 0.8344, respectively) (Table 1, Figure 5a). Native toads had the highest nucleotide diversity (*π*: 0.00469) compared to considerably lower diversity in Hawai'ian and Australian toads (*π*: 0.00010 and 0.00009, respectively) (Table 1, Figure 5b). Queensland toads had similar haplotype diversity but higher nucleotide diversity and haplotype richness to Hawai'ian toads. The population from the Northern Territory had noticeably lower genetic diversity and haplotype richness than other Australian populations (Table 1, Figure 5a,c). When the relationship between haplotypes across the invasion trajectory is considered, the 12 haplotypes from French Guiana were highly dissimilar to those from the Australian and Hawai'ian populations, forming two distinct clades (Figure 3). These were separated from each other by a minimum of 110 base pair changes and from the common invasive haplotype by a minimum of 38 base pair changes (Figure 3). Australian and Hawai'ian haplotypes were intermixed, and most of the haplotypes were one base pair different to other haplotypes, with invasive haplotypes being separated from at least one other haplotype by no more than three base pair changes.

AMOVA results showed that the overall genetic variation among populations (64.49%) was larger than the variation within populations (36.33%) (Table 2). Pairwise *F*_st_ values were significant between the native population and each introduced population (*F*_st_: 0.55–0.67; Table 3). The *F*_st_ values among introduced populations were all equal to or less than 0.05, indicating substantially less differentiation in the invasive range than in the native range. The Hawai'ian population was significantly differentiated from Australian populations overall (*F*_st_: 0.02, *p* = .02), but this difference was driven by the Northern Territory (*F*_st_: 0.05, *p* = .01) and Western Australian (*F*_st_: 0.03, *p* = .01) populations; Queensland was not significantly differentiated to the Hawai'ian population (*F*_st_: 0.01, *p* = .06). Within Australia, the Queensland population showed significant differentiation to both the Northern Territory (*F*_st_: 0.04, *p* = .01) and Western Australian (*F*_st_: 0.03, *p* = .003) populations, whereas the Northern Territory and Western Australian populations were not genetically differentiated (*F*_st_: 0, *p* = .72).

**TABLE 2:** Analysis of molecular variance (AMOVA) of all populations.

Source of variation	df	Sum of squares	% of variation	Fixation indices
All populations				
Among groups	2	482.96	64.49	
Among populations within groups	2	2.91	−0.82	
Within populations	127	579.35	36.33	*F*_ST_ = 0.64***

*** *p* < .001.

**TABLE 3:** Population differentiation (pairwise *F*_st_) between native range and introduced ranges (Hawai'i and Australia) (below the diagonal line).

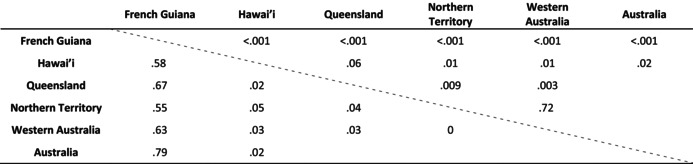

*Note*: The associated *p*‐values are shown above the diagonal line.

Two neutrality tests were implemented to study the demographic history of cane toad populations: Fu's *Fs* and Ramos‐Onsins and Rozas' *R* ^2^ statistic (Table 4). The native population had non‐significant positive Fu's *F*s values and a large *R* ^2^ statistic, indicating the population is not expanding or shrinking (i.e. is demographically stable). Fu's *F*s values were negative in all introduced populations, indicating an excess of rare haplotypes compared to the expectation from neutrality, and all these were statistically significant. *R* ^2^ statistics were significant in Hawai'i, Queensland, and Western Australia's populations.

**TABLE 4:** Demographic estimators of native‐range population (French Guiana) and introduced populations (Hawai'i and Australia).

	Fu's *Fs*	*R* ^2^	Demographic expansion		Spatial expansion	
			SSD	Raggedness	SSD	Raggedness
French Guiana	1.29 (*p* = .66)	.18 (*p* = .891)	0.03 (*p* = .19)	0.03 (*p* = .39)	0.03 (*p* = .08)	0.03 (*p* = .55)
Hawai'i	−8.78 (*p* < .001)	.08 (*p* = .02)	0.01 (*p* = .44)	0.080 (*p* = .24)	0.01 (*p* = .31)	0.08 (*p* = .21)
Australia	−27.92 (*p* < .001)	.02 (*p* < .001)	0.002 (*p* = .30)	0.067 (*p* = .16)	0.002 (*p* = .20)	0.07 (*p* = .14)
Queensland	−23.04 (*p* < .001)	.03 (*p* < .001)	0.001 (*p* = .70)	0.05 (*p* = .38)	0.002 (*p* = .66)	0.05 (*p* = .42)
Northern Territory	−3.91 (*p* < .001)	.10 (*p* = .072)	0.03 (*p* = .07)	0.21 (*p* = .03)	0.03 (*p* = .03)	0.21 (*p* = .05)
Western Australia	−8.950 (*p* < .001)	.05 (*p* = .001)	0.003 (*p* = .58)	0.08 (*p* = .34)	0.003 (*p* = .35)	0.08 (*p* = .26)

Mismatch distribution graphs of all populations are shown in Figure S1. The native population showed a multimodal distribution with non‐significant SSD and raggedness values, indicating support for spatial and demographic expansion (Table 4). Most introduced populations showed a unimodal distribution for both expansion models with non‐significant SSD and raggedness indices (Table 4), supporting the presence of spatial and demographic expansions. However, the Northern Territory population had significant or marginally significant *p*‐values for SSD and raggedness values for both expansion models indicating poor support (spatial expansion: SSD *p*‐value = .03, raggedness *p*‐value = .05; demographic expansion: SSD *p*‐value = .07, raggedness *p*‐value = .03).

## 4 DISCUSSION

Here, we constructed the first reference mitochondrial genome of *R. marina* and used nearly complete mitochondrial genome sequences to study the population genetics of the iconic cane toad in its native range and invasive ranges in Hawai'i and Australia. We utilised RNA‐Seq‐derived mitogenomes to illustrate a greater number and diversity of haplotypes in mtDNA present in Australia and Hawai'i, in contrast to the surprisingly low genetic diversity previously described using the *ND3* gene alone. We also found evidence of demographic and spatial expansion in invasive cane toad populations. This study provides new insights into the diversity of native cane toads and how they have evolved along their invasion trajectory.

### 4.1 Considerations for data validity

The use of transcriptomic data to build mitochondrial genomes has been suggested as a cost‐effective alternative to long PCRs or direct sequencing of total DNA (Forni et al., 2019), but it is important to ensure that the inferred polymorphisms accurately reflect genetic variation. In our comparison of mtDNA data obtained from WGS and RNA‐Seq, we found discrepancies in multiple nucleotide positions, all of which were in rRNA, tRNA, and the control region. These could be the result of post‐transcriptional modifications or RNA editing, which are well‐recognised phenomena across multiple taxa (Levanon et al., 2004; Porath et al., 2017; St Laurent et al., 2013). While common substitutions such as adenosine‐to‐inosine (A‐to‐I) deamination and cytidine‐to‐uridine (C‐to‐U) pyrimidine exchange were found, we also observed less common substitutions (e.g. thymine‐to‐cytidine (T‐to‐C)) that have been previously documented in another amphibian, *Xenopus tropicalis* (Zaranek et al., 2010). These processes are important for creating multiple transcript isoforms and gene regulation (Tang et al., 2012) and are known to be associated with adaptation (Duan et al., 2017, 2021). RNA editing of the tRNA anticodon recognition site would extend the codon‐matching ability and further increase the variation of the resulting translated proteins. Although we did not observe nucleotide differences in position 34 or position 37 of tRNA, which are typically recognised for deamination of adenosine to inosine, which can be paired with A, C, or U residues (Jackman & Alfonzo, 2013), we did find changes in the D arm of tRNA (aspartic acid and lysine), the T arm of tRNA (phenylalanine), and the acceptor stem of tRNA (tryptophan) (Figure S2), which are critical for the recognition of aminoacyl tRNA synthetases to bind appropriate amino acids to its designated tRNA (Ganesh & Maerkl, 2022). Failure to form a tRNA–amino acid complex could inhibit the use of a specific amino acid in subsequent protein translation. Further proteome analysis is required to determine whether these changes affect the protein translation machinery.

Post‐transcriptional modifications were more diverse in the native populations than in the invasive populations. In particular, one individual from French Guiana displayed discrepancies in the control region between WGS and RNA‐Seq data, all of which were transitions. This observation might result from tissue‐specific expression in spleen, heteroplasmy, or RNA editing. Studies of RNA editing in mtDNA have primarily focused on protein‐coding genes and tRNAs, with less research conducted on the control region of the mtDNA, which is responsible for regulating RNA and DNA synthesis. While the implications of potential RNA editing in cane toads remain unclear, these events may contribute to diversity at a transcriptional and translational level.

NUMTs can potentially elevate estimates of mitochondrial genetic variation and contribute to false‐positive signals of variation in nuclear data (Song et al., 2008). Despite NUMTs generally being perceived as non‐functional due to their ability to shift reading frames, the presence of in‐frame stop codons, and the use of different genetic codes, the transcription of NUMTs into non‐coding RNA molecules can be captured by RNA‐Seq and accidentally incorporated into assembled mitogenomes due to sequence similarity. The base differences in NUMTs will be potentially falsely interpreted as mitochondrial variation among or within individuals. Investigation of the presence/absence of NUMTs in cane toad nuclear genome and the potential expression of NUMTs is required to distinguish between genuine mtDNA variation within individuals and false‐positive signals caused by incorporating nuclear‐derived sequencing reads into mitochondrial population genetic analyses.

A search for NUMTs in the published cane toad draft genome (Edwards et al., 2018) only yielded only matches with simple repeat regions within the mtDNA control region. In other species, NUMTs originate across the entire mtDNA genome (Dayama et al., 2020; Wei et al., 2022). Following the repeat masking of the draft nuclear genome, we found no evidence of NUMTs suggesting that the putative NUMTs were just low‐complexity regions in the nuclear genome. While ancient NUMTs may be too divergent to be detected by the methods employed, NUMTs with such low identity would not interfere with our mtDNA analysis.

It is worth noting that the consensus sequences generated from the bioinformatic pipelines used in this study represented the most common nucleotide at positions where multiple nucleotides were found. Although these discrepancies could result from sequencing errors, our filtering is likely to have prevented them. As discussed above, RNA editing or NUMTs could have resulted in putative polymorphisms within individuals in our RNA‐Seq dataset. It is also possible that in some cases, heteroplasmy could have caused these putative polymorphisms. While determining the cause of this phenomenon is beyond the scope of this study, it is unlikely that this has affected population‐level statistics.

### 4.2 Population genetic analyses

Interestingly, both the haplotype network (Figure 3) and the multimodal mismatch distribution (Figure S1) indicate the presence of multiple genetically divergent lineages within native populations. Notably, individuals within each lineage are intermixed, rather than being grouped by geographical locations. For instance, despite an 80 km geographical separation, haplotypes H4 and H7 are found in the same lineage, while haplotypes H12 and H11 were collected only 9 m apart but belong to different lineages. Native‐range cane toads can travel 20–180 m daily for sheltering and foraging purposes (Shine et al., 2021). However, the overall dispersal rates are quite low, as the toads ultimately tend to return to the same shelters (DeVore et al., 2021). Consequently, the possibility of introgression between toads from these distinct lineages exists. However, a recent study in South and Central America which utilised ddRAD sequencing has offered a different perspective on the phylogenetic relationship of cane toads. This study revealed toads from the coastal area formed two sister clades, distinct from the clade formed by toads from the rainforest regions (Mittan‐Moreau et al., 2022). It becomes crucial to ascertain the precise populations of cane toads from the native range that were used as founding individuals, especially given the limited comprehensive description in prior research (Easteal, 1981). In Shine et al. (2021), cane toads from the range‐edge regions (Northern Territory and Western Australia) exhibited similar dispersal patterns to native toads in one of the coastal sites. This suggests the adaptation of cane toads in hot and arid environments is likely facilitated by the standing genetic variation inherited from the progenitors utilising dry, saline coastal beach environments (DeVore et al., 2021), rather than relying solely on the genetic adaptation and selection of novel mutation(s). A more thorough investigation into these distinct lineages becomes imperative for unravelling the mechanisms underlying the adaptations of cane toads in Australia.

Our study challenges the assumption of low genetic diversity in Hawai'ian and Australian populations. Slade and Moritz (1998) reported a unique haplotype at the *ND3* gene within Australian populations, in comparison with multiple haplotypes in native populations in Central and South America. This result supported the presence of founder effects following the introduction of cane toads into Hawai'i and Australia. With broader sampling and deeper sequencing in this study, we revealed two additional, previously undocumented, *ND3* haplotypes (Hap2 and Hap3) in Hawai'ian and Australian populations, each of which differed by only one nucleotide from the previously identified haplotype (Hap1) (Figure 4). In comparison with our analysis using nearly complete mitogenomes discussed above, this analysis highlights that a single gene is not sufficient to accurately infer genetic diversity.

The significant decrease in haplotype diversity and nucleotide diversity observed in both Hawai'ian and Australian populations, compared to native populations, does support that genetic bottlenecks and founder effects occurred post‐introduction (Table 1, Figure 5). This finding is consistent with previous studies on this species using nuclear genetic markers including SNPs, microsatellite markers, and MHC class I and II loci (Lillie et al., 2014, 2016; Selechnik, Richardson, Shine, DeVore, et al., 2019). In this study, all three neutrality indices showed strong signals of population expansion and the observed data fitted to both demographic expansion and spatial expansion models in most invasive sampling sites (Table 4). This conclusion is supported by the star‐shaped topology of the haplotype network created using the nearly complete mitochondrial genome dataset (Figure 3). Despite reduced diversity in the introduced range, the degree of the decrease is less than previously reported (Leblois et al., 2000). Additionally, the star‐shaped topology of the network containing the introduced haplotypes, the absence of native samples here, and the significant negative Fu's *F*s values indicate that many of the singleton haplotypes discovered in Hawai'i and Australia may be the products of post‐introduction mutations. Alternatively, due to the limited sample sizes in the native range, it is possible that any introduced haplotypes exist but were not included in our sample set for analysis.

We observed a curvilinear pattern of genetic diversity indices across Australian populations (from Queensland to Western Australia), a pattern also found in spleen gene expression (Selechnik, Richardson, Shine, Brown, & Rollins, 2019), limb length (Hudson, McCurry, et al., 2016), spleen, and body mass (Brown, Kelehear, Shilton, Phillips, & Shine, 2015). The mechanism behind this reduction of genetic diversity in Northern Territory toads remains unclear, but it may be due to demographic processes that reduce the effective population size and increase genetic drift (Nei & Tajima, 1981), genetic hitchhiking that removes neutral polymorphic sites under positive selection (Fay & Wu, 2000), or directional selection due to harsh climate (Selechnik, Richardson, Shine, DeVore, et al., 2019), possibly in combination with dispersion‐driven spatial sorting at the invasion front (Clarke et al., 2019). Despite our finding that the Northern Territory and Western Australian populations were not genetically differentiated, it is likely that they have experienced different local evolutionary forces and demographic processes. Northern Territory toads, unlike other populations, only exhibited demographic expansion and not spatial expansion, as supported by lower levels in all statistical measures.

Our results demonstrate significant differentiation between native and introduced populations (Table 3). We identified three genetic clusters: (1) native toads, (2) Hawai'ian and Queensland toads (*F*_st_ between locations: 0.02, *p* = .06), and (3) Northern Territory and Western Australian toads (*F*_st_ between locations: 0, *p* = .72), consistent with previous results using SNPs from the nuclear genome (Selechnik, Richardson, Shine, DeVore, et al., 2019). It is interesting that the Queensland toads are not differentiated from those sampled in Hawai'i but differ from toads sampled in the Northern Territory and Western Australia. Queensland toads have been separated from their Hawai'ian conspecifics for almost 90 years but do share similar environmental conditions (Selechnik, Richardson, Shine, DeVore, et al., 2019). Northern Territory and Western Australian toads suffer from reduced genetic diversity compared to Queensland toads, so they have likely experienced more genetic drift, which may explain this difference. However, it is also possible that the striking reduction in precipitation and increase in temperature experienced by Northern Territory and Western Australian toads compared to those from Queensland have resulted in selective differences in the mitogenome between these populations. In support of this possibility, significant negative Fu's *Fs* can indicate the presence of selection.

### 4.3 Conclusion

Our study used the whole mtDNA genome as a genetic marker to investigate the evolution of cane toads across their invasion trajectory from the native range to Australia, adding to our knowledge of changes to genetic diversity as cane toads have been serially introduced to multiple locations. Unsurprisingly, we found reduced genetic diversity in introduced populations, suggesting that genetic bottlenecks have occurred in these populations. Despite this reduction, our data indicate greater genetic diversity in introduced populations than has been previously described using mtDNA. We identified signatures of demographic and spatial expansion in most introduced localities we sampled, consistent with ongoing invasion. We described 46 haplotypes in introduced populations, mostly consisting of single base pair changes, suggesting evolution following introduction. By comparing WGS‐ and RNA‐Seq‐derived mitogenomes, we have identified the presence of post‐transcriptional modification or RNA editing, which may supply additional genetic diversity upon which selection can act.

## AUTHOR CONTRIBUTIONS

**Kelton Cheung:** Conceptualization (equal); data curation (equal); formal analysis (lead); methodology (equal); writing – original draft (lead); writing – review and editing (equal). **Timothy G. Amos:** Data curation (supporting); formal analysis (supporting); methodology (supporting); software (supporting); writing – review and editing (supporting). **Rick Shine:** Conceptualization (supporting); funding acquisition (equal); writing – review and editing (equal). **Jayna L. DeVore:** Resources (supporting); writing – review and editing (equal). **Simon Ducatez:** Resources (supporting); writing – review and editing (equal). **Richard J. Edwards:** Conceptualization (equal); data curation (equal); formal analysis (supporting); methodology (equal); software (supporting); supervision (equal); writing – original draft (supporting); writing – review and editing (equal). **Lee Ann Rollins:** Conceptualization (equal); funding acquisition (equal); project administration (lead); resources (lead); supervision (equal); writing – original draft (supporting); writing – review and editing (equal).

## Supporting information


Data S1.

## Data Availability

The reference mitochondrial genome of the cane toad (*Rhinella marina*) is available at NCBI GenBank: NC_066225.1. The code, Supporting Information, and data that support the findings of this study are available in the Open Science Framework (OSF) at https://osf.io/2ajtm/.

## References

[ece311115-bib-0001] Acevedo, A. A. , Lampo, M. , & Cipriani, R. (2016). The cane or marine toad, *Rhinella marina* (Anura, Bufonidae): Two genetically and morphologically distinct species. Zootaxa, 4103(6), 574–586. 10.11646/zootaxa.4103.6.7 27394759

[ece311115-bib-0002] Alford, R. , Brown, G. , Schwarzkopf, L. , Phillips, B. , & Shine, R. (2009). Comparisons through time and space suggest rapid evolution of dispersal behavior in an invasive species. Wildlife Research, 36, 23–28. 10.1071/wr08021

[ece311115-bib-0003] Allendorf, F. W. , & Lundquist, L. L. (2003). Introduction: Population biology, evolution, and control of invasive species. Conservation Biology, 17(1), 24–30. 10.1046/j.1523-1739.2003.02365.x

[ece311115-bib-0004] Baker, E. P. , Peris, D. , Moriarty, R. V. , Li, X. C. , Fay, J. C. , & Hittinger, C. T. (2019). Mitochondrial DNA and temperature tolerance in lager yeasts. Science Advances, 5(1), eaav1869. 10.1126/sciadv.aav1869 30729163 PMC6353617

[ece311115-bib-0005] Bandelt, H. J. , Forster, P. , & Rohl, A. (1999). Median‐joining networks for inferring intraspecific phylogenies. Molecular Biology and Evolution, 16(1), 37–48. 10.1093/oxfordjournals.molbev.a026036 10331250

[ece311115-bib-0006] Bandelt, H. J. , Forster, P. , Sykes, B. C. , & Richards, M. B. (1995). Mitochondrial portraits of human populations using median networks. Genetics, 141(2), 743–753. 10.1093/genetics/141.2.743 8647407 PMC1206770

[ece311115-bib-0007] Bernt, M. , Donath, A. , Jühling, F. , Externbrink, F. , Florentz, C. , Fritzsch, G. , Pütz, J. , Middendorf, M. , & Stadler, P. F. (2013). MITOS: Improved de novo metazoan mitochondrial genome annotation. Molecular Phylogenetics and Evolution, 69(2), 313–319. 10.1016/j.ympev.2012.08.023 22982435

[ece311115-bib-0008] Bonebrake, T. C. , Brown, C. J. , Bell, J. D. , Blanchard, J. L. , Chauvenet, A. , Champion, C. , Chen, I. C. , Clark, T. D. , Colwell, R. K. , Danielsen, F. , Dell, A. I. , Donelson, J. M. , Evengård, B. , Ferrier, S. , Frusher, S. , Garcia, R. A. , Griffis, R. B. , Hobday, A. J. , Jarzyna, M. A. , … Pecl, G. T. (2018). Managing consequences of climate‐driven species redistribution requires integration of ecology, conservation and social science. Biological Reviews, 93(1), 284–305. 10.1111/brv.12344 28568902

[ece311115-bib-0009] Bradshaw, C. J. , Leroy, B. , Bellard, C. , Roiz, D. , Albert, C. , Fournier, A. , Barbet‐Massin, M. , Salles, J. M. , Simard, F. , & Courchamp, F. (2016). Massive yet grossly underestimated global costs of invasive insects. Nature Communications, 7, 12986. 10.1038/ncomms12986 PMC5059451 27698460

[ece311115-bib-0010] Brown, G. P. , Kelehear, C. , Shilton, C. M. , Phillips, B. L. , & Shine, R. (2015). Stress and immunity at the invasion front: A comparison across cane toad (*Rhinella marina*) populations. Biological Journal of the Linnean Society, 116(4), 748–760. 10.1111/bij.12623

[ece311115-bib-0011] Brown, G. P. , Phillips, B. L. , Dubey, S. , & Shine, R. (2015). Invader immunology: Invasion history alters immune system function in cane toads (*Rhinella marina*) in tropical Australia. Ecology Letters, 18(1), 57–65. 10.1111/ele.12390 25399668

[ece311115-bib-0012] Chen, Y. , Gong, L. , Liu, X. , Chen, X. , Yang, S. , & Luo, Y. (2020). Mitochondrial DNA genomes revealed different patterns of high‐altitude adaptation in high‐altitude Tajiks compared with Tibetans and Sherpas. Scientific Reports, 10(1), 10592. 10.1038/s41598-020-67519-z 32601317 PMC7324373

[ece311115-bib-0013] Cheng, Y. T. , Liu, J. , Yang, L. Q. , Sun, C. , & Kong, Q. P. (2013). Mitochondrial DNA content contributes to climate adaptation using Chinese populations as a model. PLoS One, 8(11), e79536. 10.1371/journal.pone.0079536 24255706 PMC3821843

[ece311115-bib-0014] Chinchio, E. , Crotta, M. , Romeo, C. , Drewe, J. A. , Guitian, J. , & Ferrari, N. (2020). Invasive alien species and disease risk: An open challenge in public and animal health. PLoS Pathogens, 16(10), e1008922. 10.1371/journal.ppat.1008922 33091094 PMC7580882

[ece311115-bib-0015] Clarke, G. S. , Shine, R. , & Phillips, B. L. (2019). May the (selective) force be with you: Spatial sorting and natural selection exert opposing forces on limb length in an invasive amphibian. Journal of Evolutionary Biology, 32(9), 994–1001. 10.1111/jeb.13504 31278788

[ece311115-bib-0016] Davey, N. E. , Shields, D. C. , & Edwards, R. J. (2006). SLiMDisc: Short, linear motif discovery, correcting for common evolutionary descent. Nucleic Acids Research, 34(12), 3546–3554. 10.1093/nar/gkl486 16855291 PMC1524906

[ece311115-bib-0017] Dayama, G. , Zhou, W. , Prado‐Martinez, J. , Marques‐Bonet, T. , & Mills, R. E. (2020). Characterization of nuclear mitochondrial insertions in the whole genomes of primates. NAR Genomics and Bioinformatics, 2(4), lqaa089. 10.1093/nargab/lqaa089 33575633 PMC7671390

[ece311115-bib-0018] DeVore, J. L. , Shine, R. , & Ducatez, S. (2021). Spatial ecology of cane toads (*Rhinella marina*) in their native range: A radiotelemetric study from French Guiana. Scientific Reports, 11(1), 11817. 10.1038/s41598-021-91262-8 34083703 PMC8175754

[ece311115-bib-0019] Dobin, A. , Davis, C. A. , Schlesinger, F. , Drenkow, J. , Zaleski, C. , Jha, S. , Batut, P. , Chaisson, M. , & Gingeras, T. R. (2013). STAR: ultrafast universal RNA‐seq aligner. Bioinformatics, 29(1), 15–21. 10.1093/bioinformatics/bts635 23104886 PMC3530905

[ece311115-bib-0020] Duan, Y. , Dou, S. , Luo, S. , Zhang, H. , & Lu, J. (2017). Adaptation of A‐to‐I RNA editing in Drosophila. PLoS Genetics, 13(3), e1006648. 10.1371/journal.pgen.1006648 28282384 PMC5365144

[ece311115-bib-0021] Duan, Y. , Dou, S. , Porath, H. T. , Huang, J. , Eisenberg, E. , & Lu, J. (2021). A‐to‐I RNA editing in honeybees shows signals of adaptation and convergent evolution. iScience, 24(1), 101983. 10.1016/j.isci.2020.101983 33458624 PMC7797907

[ece311115-bib-0022] Easteal, S. (1981). The history of introductions of *Bufo marinus* (Amphibia: Anura); a natural experiment in evolution. Biological Journal of the Linnean Society, 16(2), 93–113. 10.1111/j.1095-8312.1981.tb01645.x

[ece311115-bib-0023] Edwards, R. J. , Field, M. A. , Ferguson, J. M. , Dudchenko, O. , Keilwagen, J. , Rosen, B. D. , Johnson, G. S. , Rice, E. S. , Hillier, L. D. , Hammond, J. M. , Towarnicki, S. G. , Omer, A. , Khan, R. , Skvortsova, K. , Bogdanovic, O. , Zammit, R. A. , Aiden, E. L. , Warren, W. C. , & Ballard, J. W. O. (2021). Chromosome‐length genome assembly and structural variations of the primal Basenji dog (*Canis lupus familiaris*) genome. BMC Genomics, 22(1), 188. 10.1186/s12864-021-07493-6 33726677 PMC7962210

[ece311115-bib-0024] Edwards, R. J. , Paulsen, K. , Aguilar Gomez, C. M. , & Perez‐Bercoff, A. (2020). Computational prediction of disordered protein motifs using SLiMSuite. Methods in Molecular Biology, 2141, 37–72. 10.1007/978-1-0716-0524-0_3 32696352

[ece311115-bib-0025] Edwards, R. J. , Tuipulotu, D. E. , Amos, T. G. , O'Meally, D. , Richardson, M. F. , Russell, T. L. , Vallinoto, M. , Carneiro, M. , Ferrand, N. , Wilkins, M. R. , Sequeira, F. , Rollins, L. A. , Holmes, E. C. , Shine, R. , & White, P. A. (2018). Draft genome assembly of the invasive cane toad, *Rhinella marina* . GigaScience, 7(9), giy095. 10.1093/gigascience/giy095 30101298 PMC6145236

[ece311115-bib-0026] Estoup, A. , Ravigné, V. , Hufbauer, R. , Vitalis, R. , Gautier, M. , & Facon, B. (2016). Is there a genetic paradox of biological invasion? Annual Review of Ecology, Evolution, and Systematics, 47(1), 51–72. 10.1146/annurev-ecolsys-121415-032116

[ece311115-bib-0027] Excoffier, L. (2004). Patterns of DNA sequence diversity and genetic structure after a range expansion: Lessons from the infinite‐Island model. Molecular Ecology, 13(4), 853–864. 10.1046/j.1365-294x.2003.02004.x 15012760

[ece311115-bib-0028] Excoffier, L. , & Lischer, H. E. (2010). Arlequin suite ver 3.5: A new series of programs to perform population genetics analyses under Linux and windows. Molecular Ecology Resources, 10(3), 564–567. 10.1111/j.1755-0998.2010.02847.x 21565059

[ece311115-bib-0029] Fay, J. C. , & Wu, C. I. (2000). Hitchhiking under positive Darwinian selection. Genetics, 155(3), 1405–1413. 10.1093/genetics/155.3.1405 10880498 PMC1461156

[ece311115-bib-0030] Forni, G. , Puccio, G. , Bourguignon, T. , Evans, T. , Mantovani, B. , Rota‐Stabelli, O. , & Luchetti, A. (2019). Complete mitochondrial genomes from transcriptomes: Assessing pros and cons of data mining for assembling new mitogenomes. Scientific Reports, 9(1), 14806. 10.1038/s41598-019-51313-7 31616005 PMC6794255

[ece311115-bib-0031] Fu, Y. X. (1997). Statistical tests of neutrality of mutations against population growth, hitchhiking and background selection. Genetics, 147(2), 915–925. 10.1093/genetics/147.2.915 9335623 PMC1208208

[ece311115-bib-0032] Ganesh, R. B. , & Maerkl, S. J. (2022). Biochemistry of aminoacyl tRNA Synthetase and tRNAs and their engineering for cell‐free and synthetic cell applications. Frontiers in Bioengineering and Biotechnology, 10, 918659. 10.3389/fbioe.2022.918659 35845409 PMC9283866

[ece311115-bib-0033] Goudet, J. (1995). FSTAT (version 1.2): A computer program to calculate F‐statistics. Journal of Heredity, 86(6), 485–486. 10.1093/oxfordjournals.jhered.a111627

[ece311115-bib-0034] Gravel, S. (2016). When is selection effective? Genetics, 203(1), 451–462. 10.1534/genetics.115.184630 27010021 PMC4858791

[ece311115-bib-0035] Greiner, S. , Lehwark, P. , & Bock, R. (2019). OrganellarGenomeDRAW (OGDRAW) version 1.3.1: Expanded toolkit for the graphical visualization of organellar genomes. Nucleic Acids Research, 47(W1), W59–W64. 10.1093/nar/gkz238 30949694 PMC6602502

[ece311115-bib-0036] Gruber, J. , Brown, G. , Whiting, M. J. , & Shine, R. (2017). Is the behavioural divergence between range‐core and range‐edge populations of cane toads (*Rhinella marina*) due to evolutionary change or developmental plasticity? Royal Society Open Science, 4(10), 170789. 10.1098/rsos.170789 29134082 PMC5666265

[ece311115-bib-0037] Gruber, J. , Brown, G. , Whiting, M. J. , & Shine, R. (2018). Behavioural divergence during biological invasions: A study of cane toads (*Rhinella marina*) from contrasting environments in Hawai'i. Royal Society Open Science, 5(4), 180197. 10.1098/rsos.180197 29765696 PMC5936961

[ece311115-bib-0038] Gruber, J. , Whiting, M. J. , Brown, G. , & Shine, R. (2017). The loneliness of the long‐distance toad: Invasion history and social attraction in cane toads (*Rhinella marina*). Biology Letters, 13(11), 20170445. 10.1098/rsbl.2017.0445 29118242 PMC5719377

[ece311115-bib-0039] Hudson, C. M. , Brown, G. P. , & Shine, R. (2016). It is lonely at the front: Contrasting evolutionary trajectories in male and female invaders. Royal Society Open Science, 3(12), 160687. 10.1098/rsos.160687 28083108 PMC5210690

[ece311115-bib-0040] Hudson, C. M. , McCurry, M. R. , Lundgren, P. , McHenry, C. R. , & Shine, R. (2016). Constructing an invasion machine: The rapid evolution of a dispersal‐enhancing phenotype during the cane toad invasion of Australia. PLoS One, 11(9), e0156950. 10.1371/journal.pone.0156950 27658247 PMC5033235

[ece311115-bib-0041] Jackman, J. E. , & Alfonzo, J. D. (2013). Transfer RNA modifications: nature's combinatorial chemistry playground. Wiley Interdisciplinary Reviews: RNA, 4(1), 35–48. 10.1002/wrna.1144 23139145 PMC3680101

[ece311115-bib-0042] Katoh, K. , & Standley, D. M. (2013). MAFFT multiple sequence alignment software version 7: Improvements in performance and usability. Molecular Biology and Evolution, 30(4), 772–780. 10.1093/molbev/mst010 23329690 PMC3603318

[ece311115-bib-0043] Klucnika, A. , & Ma, H. (2019). A battle for transmission: The cooperative and selfish animal mitochondrial genomes. Open Biology, 9(3), 180267. 10.1098/rsob.180267 30890027 PMC6451365

[ece311115-bib-0044] Langmead, B. , & Salzberg, S. L. (2012). Fast gapped‐read alignment with Bowtie 2. Nature Methods, 9(4), 357–359. 10.1038/nmeth.1923 22388286 PMC3322381

[ece311115-bib-0045] Larsson, A. (2014). AliView: A fast and lightweight alignment viewer and editor for large datasets. Bioinformatics, 30(22), 3276–3278. 10.1093/bioinformatics/btu531 25095880 PMC4221126

[ece311115-bib-0046] Leblois, R. , Rousset, F. , Tikel, D. , Moritz, C. , & Estoup, A. (2000). Absence of evidence for isolation by distance in an expanding cane toad (*Bufo marinus*) population: An individual‐based analysis of microsatellite genotypes. Molecular Ecology, 9(11), 1905–1909. 10.1046/j.1365-294x.2000.01091.x 11091326

[ece311115-bib-0047] Levanon, E. Y. , Eisenberg, E. , Yelin, R. , Nemzer, S. , Hallegger, M. , Shemesh, R. , Fligelman, Z. Y. , Shoshan, A. , Pollock, S. R. , Sztybel, D. , Olshansky, M. , Rechavi, G. , & Jantsch, M. F. (2004). Systematic identification of abundant A‐to‐I editing sites in the human transcriptome. Nature Biotechnology, 22(8), 1001–1005. 10.1038/nbt996 15258596

[ece311115-bib-0048] Lillie, M. , Cui, J. , Shine, R. , & Belov, K. (2016). Molecular characterization of MHC class II in the Australian invasive cane toad reveals multiple splice variants. Immunogenetics, 68(6–7), 449–460. 10.1007/s00251-016-0919-9 27233954

[ece311115-bib-0049] Lillie, M. , Dubey, S. , Shine, R. , & Belov, K. (2017). Variation in major histocompatibility complex diversity in invasive cane toad populations. Wildlife Research, 44(7), 565. 10.1071/wr17055

[ece311115-bib-0050] Lillie, M. , Shine, R. , & Belov, K. (2014). Characterisation of major histocompatibility complex class I in the Australian cane toad, *Rhinella marina* . PLoS One, 9(8), e102824. 10.1371/journal.pone.0102824 25093458 PMC4122387

[ece311115-bib-0051] Lowe, S. , Browne, M. , Boudjelas, S. , & De Poorter, M. (2000). 100 of the World's Worst Invasive Alien Species: A Selection From the Global Invasive Species Database. *Published by The Invasive Species Specialist Group (ISSG) a specialist group of the Species Survival Commission (SSC) of the World Conservation Union (IUCN), 12pp. First published as special lift‐out in Aliens, 12* .

[ece311115-bib-0052] Machado, D. J. , Lyra, M. L. , & Grant, T. (2016). Mitogenome assembly from genomic multiplex libraries: Comparison of strategies and novel mitogenomes for five species of frogs. Molecular Ecology Resources, 16(3), 686–693. 10.1111/1755-0998.12492 26607054

[ece311115-bib-0053] Mainka, S. A. , & Howard, G. W. (2010). Climate change and invasive species: Double jeopardy. Integrative Zoology, 5(2), 102–111. 10.1111/j.1749-4877.2010.00193.x 21392328

[ece311115-bib-0054] McGaughran, A. , Dhami, M. K. , Parvizi, E. , Vaughan, A. L. , Gleeson, D. M. , Hodgins, K. A. , Rollins, L. A. , Tepolt, C. K. , Turner, K. G. , Atsawawaranunt, K. , Battlay, P. , Congrains, C. , Crottini, A. , Dennis, T. P. W. , Lange, C. , Liu, X. P. , Matheson, P. , North, H. L. , Popovic, I. , … Wilson, J. (2024). Genomic tools in biological invasions: Current state and future Frontiers. Genome Biology and Evolution, 16(1), evad230. 10.1093/gbe/evad230 38109935 PMC10776249

[ece311115-bib-0055] McKenna, A. , Hanna, M. , Banks, E. , Sivachenko, A. , Cibulskis, K. , Kernytsky, A. , Garimella, K. , Altshuler, D. , Gabriel, S. , Daly, M. , & DePristo, M. A. (2010). The genome analysis toolkit: A MapReduce framework for analyzing next‐generation DNA sequencing data. Genome Research, 20(9), 1297–1303. 10.1101/gr.107524.110 20644199 PMC2928508

[ece311115-bib-0056] Meiklejohn, C. D. , Montooth, K. L. , & Rand, D. M. (2007). Positive and negative selection on the mitochondrial genome. Trends in Genetics, 23(6), 259–263. 10.1016/j.tig.2007.03.008 17418445

[ece311115-bib-0057] Mittan‐Moreau, C. S. , Kelehear, C. , Toledo, L. F. , Bacon, J. , Guayasamin, J. M. , Snyder, A. , & Zamudio, K. R. (2022). Cryptic lineages and standing genetic variation across independent cane toad introductions. Molecular Ecology, 31(24), 6440–6456. 10.1111/mec.16713 36198047 PMC10091960

[ece311115-bib-0058] Nei, M. , & Tajima, F. (1981). Genetic drift and estimation of effective population size. Genetics, 98(3), 625–640. 10.1093/genetics/98.3.625 17249104 PMC1214463

[ece311115-bib-0059] Phillips, B. L. , Brown, G. P. , & Shine, R. (2010). Evolutionarily accelerated invasions: The rate of dispersal evolves upwards during the range advance of cane toads. Journal of Evolutionary Biology, 23(12), 2595–2601. 10.1111/j.1420-9101.2010.02118.x 20939838

[ece311115-bib-0060] Polzin, T. , & Vahdati Daneshmand, S. (2003). On Steiner trees and minimum spanning trees in hypergraphs. Operations Research Letters, 31, 12–20. 10.1016/S0167-6377(02)00185-2

[ece311115-bib-0061] Poplin, R. , Ruano‐Rubio, V. , DePristo, M. A. , Fennell, T. J. , Carneiro, M. O. , Van der Auwera, G. A. , Kling, D. E. , Gauthier, L. D. , Levy‐Moonshine, A. , Roazen, D. , Shakir, K. , Thibault, J. , Chandran, S. , Whelan, C. , Lek, M. , Gabriel, S. , Daly, M. J. , Neale, B. , MacArthur, D. G. , & Banks, E. (2018). Scaling accurate genetic variant discovery to tens of thousands of samples. *bioRxiv* . 10.1101/201178

[ece311115-bib-0062] Porath, H. T. , Knisbacher, B. A. , Eisenberg, E. , & Levanon, E. Y. (2017). Massive A‐to‐I RNA editing is common across the Metazoa and correlates with dsRNA abundance. Genome Biology, 18(1), 185. 10.1186/s13059-017-1315-y 28969707 PMC5625713

[ece311115-bib-0063] Radford, I. J. , Woolley, L.‐A. , Dickman, C. R. , Corey, B. , Trembath, D. , & Fairman, R. (2019). Invasive species‐driven trophic cascades: Are cane toads indirectly contributing to small mammal collapses across tropical Australia? *bioRxiv* . 10.1101/616771

[ece311115-bib-0064] Ramirez‐Soriano, A. , Ramos‐Onsins, S. E. , Rozas, J. , Calafell, F. , & Navarro, A. (2008). Statistical power analysis of neutrality tests under demographic expansions, contractions and bottlenecks with recombination. Genetics, 179(1), 555–567. 10.1534/genetics.107.083006 18493071 PMC2390632

[ece311115-bib-0065] Ramos‐Onsins, S. E. , & Rozas, J. (2002). Statistical properties of new neutrality tests against population growth. Molecular Biology and Evolution, 19(12), 2092–2100. 10.1093/oxfordjournals.molbev.a004034 12446801

[ece311115-bib-0066] Richardson, M. F. , Sequeira, F. , Selechnik, D. , Carneiro, M. , Vallinoto, M. , Reid, J. G. , West, A. J. , Crossland, M. R. , Shine, R. , & Rollins, L. A. (2018). Improving amphibian genomic resources: A multitissue reference transcriptome of an iconic invader. GigaScience, 7(1), 1–7. 10.1093/gigascience/gix114 PMC5765561 29186423

[ece311115-bib-0067] Rollins, L. A. , Moles, A. T. , Lam, S. , Buitenwerf, R. , Buswell, J. M. , Brandenburger, C. R. , Flores‐Moreno, H. , Nielsen, K. B. , Couchman, E. , Brown, G. S. , Thomson, F. J. , Hemmings, F. , Frankham, R. , & Sherwin, W. B. (2013). High genetic diversity is not essential for successful introduction. Ecology and Evolution, 3(13), 4501–4517. 10.1002/ece3.824 24340190 PMC3856749

[ece311115-bib-0068] Rollins, L. A. , Richardson, M. F. , & Shine, R. (2015). A genetic perspective on rapid evolution in cane toads (*Rhinella marina*). Molecular Ecology, 24(9), 2264–2276. 10.1111/mec.13184 25894012

[ece311115-bib-0069] Rozas, J. , Ferrer‐Mata, A. , Sánchez‐DelBarrio, J. C. , Guirao‐Rico, S. , Librado, P. , Ramos‐Onsins, S. E. , & Sánchez‐Gracia, A. (2017). DnaSP 6: DNA sequence polymorphism analysis of large data sets. Molecular Biology and Evolution, 34(12), 3299–3302. 10.1093/molbev/msx248 29029172

[ece311115-bib-0070] Sakai, A. K. , Allendorf, F. W. , Holt, J. S. , Lodge, D. M. , Molofsky, J. , With, K. A. , Baughman, S. , Cabin, R. J. , Cohen, J. E. , Ellstrand, N. C. , McCauley, D. E. , O'Neil, P. , Parker, I. M. , Thompson, J. N. , & Weller, S. G. (2001). The population biology of invasive species. Annual Review of Ecology and Systematics, 32(1), 305–332. 10.1146/annurev.ecolsys.32.081501.114037

[ece311115-bib-0071] Schneider, S. , & Excoffier, L. (1999). Estimation of past demographic parameters from the distribution of pairwise differences when the mutation rates vary among sites: Application to human mitochondrial DNA. Genetics, 152(3), 1079–1089. 10.1093/genetics/152.3.1079 10388826 PMC1460660

[ece311115-bib-0072] Schrieber, K. , & Lachmuth, S. (2017). The genetic paradox of invasions revisited: The potential role of inbreeding x environment interactions in invasion success. Biological Reviews of the Cambridge Philosophical Society, 92(2), 939–952. 10.1111/brv.12263 27009691

[ece311115-bib-0073] Seebens, H. , Blackburn, T. M. , Dyer, E. E. , Genovesi, P. , Hulme, P. E. , Jeschke, J. M. , Pagad, S. , Pyšek, P. , Winter, M. , Arianoutsou, M. , Bacher, S. , Blasius, B. , Brundu, G. , Capinha, C. , Celesti‐Grapow, L. , Dawson, W. , Dullinger, S. , Fuentes, N. , Jäger, H. , … Essl, F. (2017). No saturation in the accumulation of alien species worldwide. Nature Communications, 8, 14435. 10.1038/ncomms14435 PMC5316856 28198420

[ece311115-bib-0074] Selechnik, D. , Richardson, M. F. , Shine, R. , Brown, G. P. , & Rollins, L. A. (2019). Immune and environment‐driven gene expression during invasion: An eco‐immunological application of RNA‐Seq. Ecology and Evolution, 9(11), 6708–6721. 10.1002/ece3.5249 31236254 PMC6580278

[ece311115-bib-0075] Selechnik, D. , Richardson, M. F. , Shine, R. , DeVore, J. L. , Ducatez, S. , & Rollins, L. A. (2019). Increased adaptive variation despite reduced overall genetic diversity in a rapidly adapting invader. Frontiers in Genetics, 10, 1221. 10.3389/fgene.2019.01221 31850072 PMC6901984

[ece311115-bib-0076] Shine, R. (2010). The ecological impact of invasive cane toads (*Bufo marinus*) in Australia. The Quarterly Review of Biology, 85(3), 253–291. 10.1086/655116 20919631

[ece311115-bib-0077] Shine, R. , Alford, R. A. , Blennerhasset, R. , Brown, G. P. , DeVore, J. L. , Ducatez, S. , Finnerty, P. , Greenlees, M. , Kaiser, S. W. , McCann, S. , Pettit, L. , Pizzatto, L. , Schwarzkopf, L. , Ward‐Fear, G. , & Phillips, B. L. (2021). Increased rates of dispersal of free‐ranging cane toads (*Rhinella marina*) during their global invasion. Scientific Reports, 11(1), 23574. 10.1038/s41598-021-02828-5 34876612 PMC8651681

[ece311115-bib-0078] Shine, R. , Brown, G. P. , & Phillips, B. L. (2011). An evolutionary process that assembles phenotypes through space rather than through time. Proceedings of the National Academy of Sciences of the United States of America, 108(14), 5708–5711. 10.1073/pnas.1018989108 21436040 PMC3078378

[ece311115-bib-0079] Silva, G. , Lima, F. P. , Martel, P. , & Castilho, R. (2014). Thermal adaptation and clinal mitochondrial DNA variation of European anchovy. Proceedings of the Biological Sciences, 281(1792), 20141093. 10.1098/rspb.2014.1093 PMC4150322 25143035

[ece311115-bib-0080] Slade, R. W. , & Moritz, C. (1998). Phylogeography of Bufo marinus from its natural and introduced ranges. Proceedings of the Biological Sciences, 265(1398), 769–777. 10.1098/rspb.1998.0359 PMC1689048 9628036

[ece311115-bib-0081] Song, H. , Buhay, J. E. , Whiting, M. F. , & Crandall, K. A. (2008). Many species in one: DNA barcoding overestimates the number of species when nuclear mitochondrial pseudogenes are coamplified. Proceedings of the National Academy of Sciences of the United States of America, 105(36), 13486–13491. 10.1073/pnas.0803076105 18757756 PMC2527351

[ece311115-bib-0082] St Laurent, G. , Tackett, M. R. , Nechkin, S. , Shtokalo, D. , Antonets, D. , Savva, Y. A. , Maloney, R. , Kapranov, P. , Lawrence, C. E. , & Reenan, R. A. (2013). Genome‐wide analysis of A‐to‐I RNA editing by single‐molecule sequencing in Drosophila. Nature Structural & Molecular Biology, 20(11), 1333–1339. 10.1038/nsmb.2675 24077224

[ece311115-bib-0083] Tang, W. , Fei, Y. , & Page, M. (2012). Biological significance of RNA editing in cells. Molecular Biotechnology, 52(1), 91–100. 10.1007/s12033-012-9498-7 22271460

[ece311115-bib-0084] Tarailo‐Graovac, M. , & Chen, N. (2009). Using RepeatMasker to identify repetitive elements in genomic sequences. Current Protocols in Bioinformatics, Chapter 4, 4 10 11–14 10 14. 10.1002/0471250953.bi0410s25 19274634

[ece311115-bib-0085] Toews, D. P. , & Brelsford, A. (2012). The biogeography of mitochondrial and nuclear discordance in animals. Molecular Ecology, 21(16), 3907–3930. 10.1111/j.1365-294X.2012.05664.x 22738314

[ece311115-bib-0086] Turvey, N. (2013). Cane toads: A tale of sugar, politics and flawed science. Sydney University Press.

[ece311115-bib-0087] Urban, M. C. , Phillips, B. L. , Skelly, D. K. , & Shine, R. (2008). A toad more traveled: The heterogeneous invasion dynamics of cane toads in Australia. The American Naturalist, 171(3), E134–E148. 10.1086/527494 18271722

[ece311115-bib-0088] Van der Auwera, G. A. (2020). Genomics in the cloud: Using Docker, GATK, and WDL in Terra (1st ed.). O'Reilly Media.

[ece311115-bib-0089] Walker, B. J. , Abeel, T. , Shea, T. , Priest, M. , Abouelliel, A. , Sakthikumar, S. , Cuomo, C. A. , Zeng, Q. , Wortman, J. , Young, S. K. , & Earl, A. M. (2014). Pilon: An integrated tool for comprehensive microbial variant detection and genome assembly improvement. PLoS One, 9(11), e112963. 10.1371/journal.pone.0112963 25409509 PMC4237348

[ece311115-bib-0090] Wang, X. , Zhou, S. , Wu, X. , Wei, Q. , Shang, Y. , Sun, G. , Mei, X. , Dong, Y. , Sha, W. , & Zhang, H. (2021). High‐altitude adaptation in vertebrates as revealed by mitochondrial genome analyses. Ecology and Evolution, 11(21), 15077–15084. 10.1002/ece3.8189 34765161 PMC8571627

[ece311115-bib-0091] Wei, W. , Schon, K. R. , Elgar, G. , Orioli, A. , Tanguy, M. , Giess, A. , Tischkowitz, M. , Caulfield, M. J. , & Chinnery, P. F. (2022). Nuclear‐embedded mitochondrial DNA sequences in 66,083 human genomes. Nature, 611(7934), 105–114. 10.1038/s41586-022-05288-7 36198798 PMC9630118

[ece311115-bib-0092] Wright, E. S. (2016). Using DECIPHER v2.0 to analyze big biological sequence data in R. R Journal, 8, 352–359.

[ece311115-bib-0093] Wright, E. S. (2020). RNAconTest: Comparing tools for noncoding RNA multiple sequence alignment based on structural consistency. RNA, 26(5), 531–540. 10.1261/rna.073015.119 32005745 PMC7161358

[ece311115-bib-0094] Xiao, S. , Nguyen, D. T. , Wu, B. , & Hao, W. (2017). Genetic drift and Indel mutation in the evolution of yeast mitochondrial genome size. Genome Biology and Evolution, 9(11), 3088–3099. 10.1093/gbe/evx232 29126284 PMC5714193

[ece311115-bib-0095] Yagound, B. , West, A. J. , Richardson, M. F. , Selechnik, D. , Shine, R. , & Rollins, L. A. (2022). Brain transcriptome analysis reveals gene expression differences associated with dispersal behaviour between range‐front and range‐core populations of invasive cane toads in Australia. Molecular Ecology, 31(6), 1700–1715. 10.1111/mec.16347 35028988 PMC9303232

[ece311115-bib-0096] Zaranek, A. W. , Levanon, E. Y. , Zecharia, T. , Clegg, T. , & Church, G. M. (2010). A survey of genomic traces reveals a common sequencing error, RNA editing, and DNA editing. PLoS Genetics, 6(5), e1000954. 10.1371/journal.pgen.1000954 20531933 PMC2873906

[ece311115-bib-0097] Zug, G. R. , & Zug, P. B. (1979). The marine toad, *Bufo marinus*: A natural history resumé of native populations. Smithsonian Libraries, 284, 1–58. 10.5479/si.00810282.284

