# Supplementary material for: Whole‐mitogenome analysis unveils previously undescribed genetic diversity in cane toads across their invasion trajectory
Source: Ecol Evol. 2024 Mar 3;14(3):e11115. doi: 10.1002/ece3.11115 (PMC10909579; doi:10.1002/ece3.11115)
Supplement: Supplementary file 1 — Data S1. [file ECE3-14-e11115-s001.zip › Whole mitogenomes analysis unveils previously undescribed genetic diversity in cane toads across their invasion trajectory_Supplementary Information.pdf]

Supplemental Information for:

Whole mitogenomes analysis unveils previously undescribed genetic diversity in cane toads across their invasion trajectory

Cheung K, Amos TG, Shine R, DeVore JL, Ducatez S, Edwards RJ & Rollins LA

Table of Contents:

**Figure S1**	Page 2
**Figure S2**	Page 3
**Table S1**	Page 4-5
**Table S2**	Page 6
**Table S3**	Page 7
**Table S4**	Page 8

Figure S1: Mismatch distribution graphs of native and introduced populations. The x-axis represents the number of pairwise nucleotide differences between pairs of samples. The y-axis represents the frequency of the comparisons. Both the demographic expansion model (solid red line) and the spatial expansion model (solid blue line) were investigated.

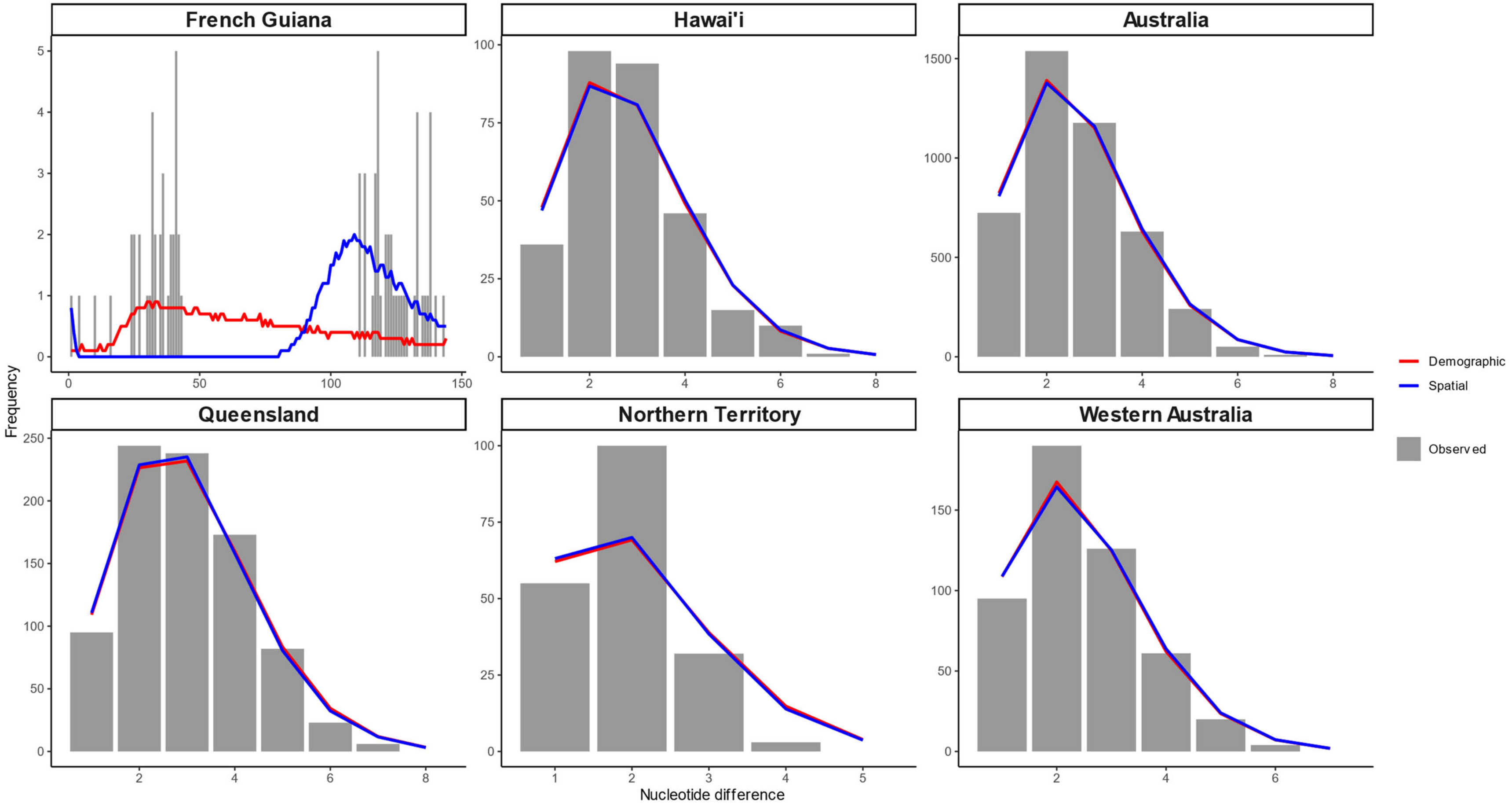

Figure S2: Predicted tRNA secondary structures showing nucleotide substitutions between WGS and RNASeq data. Nucleotides marked with red circles indicate differences between the WGS and RNASeq datasets. (a) tRNA (Lysine), (b) tRNA (Aspartic acid), (c) tRNA (Tryptophan), and (d) tRNA (Phenylalanine) from the WGS dataset (left) and the RNASeq dataset (right).

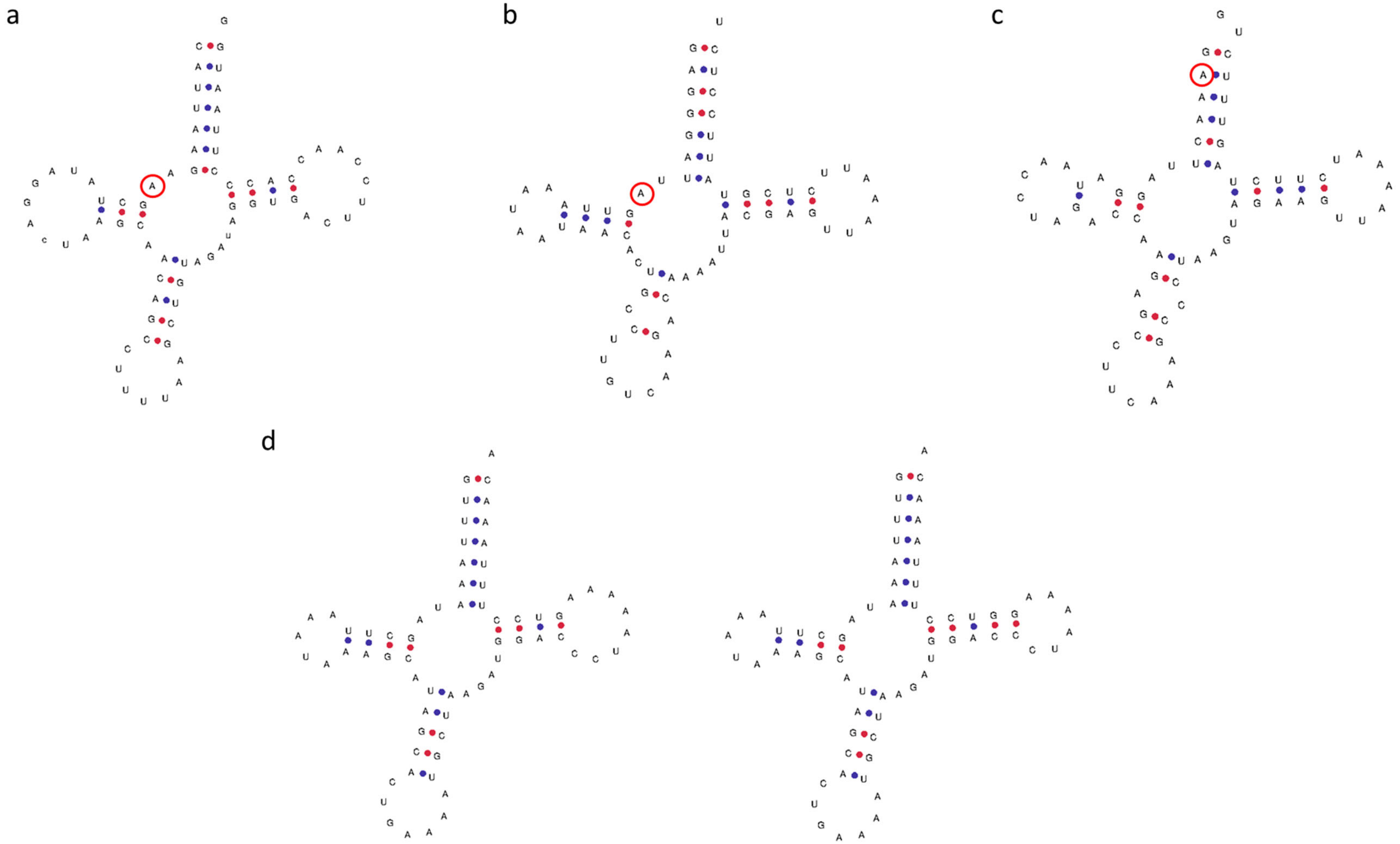

Table S1: ***Rhinella marina*** **samples used in this study.**

Site	Location	Sample ID	Tissue	Type of sequencing	Haplotype notation	Latitude	Longitude	Reference
**French Guiana**								
	Plage de Montjoly	RMF010	Spleen	RNA	H7	4.913	-52.261	
	Plage de Montjoly	RMF022	Spleen	RNA	H9	4.913	-52.267	
	Plage de Montjoly	RMF044	Liver	WGS	H3	4.913	-52.260	
	Plage de Montjoly	RMF046	Spleen	RNA	H11	4.913	-52.259	
	Plage de Montjoly	RMF047	Spleen	RNA	H12	4.913	-52.259	
	Plage de Montjoly	RMF048	Liver	RNA, WGS	H11	4.913	-52.259	
	Plage de Montjoly	RMF049	Spleen	RNA	H13	4.913	-52.260	This study
	Plage de Gosselin	RMF017	Spleen	RNA	H8	4.891	-52.253	
	Plage de Gosselin	RMF031	Liver	WGS	H1	4.890	-52.252	
	Route des Plages Drain	RMF028	Spleen	RNA	H10	4.879	-52.246	
	Past Regina wetland	RMF042	Liver	WGS	H2	4.203	-52.126	
	Past Regina wetland	RMFG0403	Toe	WGS	H4	4.203	-52.126	
	Regina Wash	RMFG107	Toe	WGS	H5	4.363	-52.280	
	Lake Maracaibo, Venezuela							
	Higuerote, Venezuela							
	Guiria, Venezuela							
	Cayenne, French Guiana							
	Madre de Dios, Atalaya, Peru							Slade and Moritz et al.
	Mexico							
	Costa Rica							
	Panama							
	Texas, USA							
**Hawai'ian Islands**								
O'ahu								
	Haiku Gardens	RMH004	Spleen	WGS	H6	21.415	-157.816	
	Haiku Gardens	RMH006	Muscle	RNA, WGS	H14	21.415	-157.816	
	Haiku Gardens	RMH008	Brain	RNA	H15	21.415	-157.816	
	Haiku Gardens	RMH043	Brain	RNA	H20	21.415	-157.816	
	Haiku Gardens	RMH044	Brain	RNA	H14	21.415	-157.816	
	Haiku Gardens	RMH046	Brain	RNA	H6	21.415	-157.816	
	Haiku Gardens	RMH047	Brain	RNA	H14	21.415	-157.816	
	Haiku Gardens	RMH049	Spleen	RNA	H21	21.415	-157.816	
	Haiku Gardens	RMH050	Spleen	RNA	H14	21.415	-157.816	This study
	Kapolei Regional Park	RMH018	Spleen	RNA	H6	21.336	-158.078	
	Kapolei Regional Park	RMH020	Brain	RNA	H6	21.336	-158.078	
	Kapolei Regional Park	RMH021	Brain	RNA	H16	21.336	-158.078	
	Kapolei Regional Park	RMH024	Spleen	RNA	H17	21.336	-158.078	
	Kapolei Regional Park	RMH025	Spleen	RNA	H18	21.336	-158.078	
	Kapolei Regional Park	RMH026	Brain	RNA	H6	21.336	-158.078	
	Kapolei Regional Park	RMH027	Brain	RNA	H6	21.336	-158.078	
	Kapolei Regional Park	RMH028	Brain	RNA	H19	21.336	-158.078	
	Manoa							Slade and Moritz et al.
Hawai'i								
	Paradise Park	RMH051	Muscle	RNA, WGS	H23	19.572	-154.958	
	Paradise Park	RMH052	Muscle	RNA, WGS	H6	19.572	-154.958	
	Paradise Park	RMH059	Brain	RNA	H22	19.572	-154.958	
	Paradise Park	RMH061	Brain	RNA	H22	19.572	-154.958	This study
	Kingslake	RMH075	Brain	RNA	H24	19.939	-155.875	
	Kingslake	RMH077	Brain	RNA	H6	19.939	-155.875	
	Kingslake	RMH079	Brain	RNA	H24	19.939	-155.875	
	Kingslake	RMH081	Brain	RNA	H25	19.939	-155.875	
	Coconut Island							Slade and Moritz et al.
**Australia**								
Western Australia								
	Caroline Pool	RM640	Brain	RNA	H21	-18.227	127.760	
	Caroline Pool	RM642	Spleen	RNA	H6	-18.227	127.760	
	Caroline Pool	RM645	Brain	RNA	H21	-18.227	127.760	
	Caroline Pool	RM647	Brain	RNA	H21	-18.227	127.760	
	Caroline Pool	RM650	Brain	RNA	H6	-18.227	127.760	
	Durack River	RM657	Brain	RNA	H52	-15.640	127.234	
	Durack River	RM658	Brain	RNA	H53	-15.640	127.234	
	Durack River	RM670	Brain	RNA	H6	-15.640	127.234	
	Durack River	RM676	Brain	RNA	H54	-15.640	127.234	
	Durack River	RM684	Spleen	RNA	H21	-15.640	127.234	
	Emma Gorge	RM010	Muscle	RNA, WGS	H33	-15.906	128.128	
	Emma Gorge	RM021	Muscle	RNA	H6	-15.906	128.128	
	Emma Gorge	RM034	Muscle	RNA	H6	-15.906	128.128	
	Emma Gorge	RM039	Muscle	RNA	H50	-15.906	128.128	
	Emma Gorge	RM045	Muscle	RNA, WGS	H51	-15.906	128.128	
	Purnululu	RM061	Muscle	RNA	H6	-17.430	128.304	
	Purnululu	RM082	Muscle	RNA	H6	-17.430	128.304	This study
	Purnululu	RM087	Muscle	RNA	H21	-17.430	128.304	
	Purnululu	RM092	Muscle	RNA	H6	-17.430	128.304	
	Purnululu	RM094	Muscle	RNA	H6	-17.430	128.304	
	Purnululu	RMP028	Brain	RNA	H16	-17.448	128.547	
	Purnululu	RMP029	Brain	RNA	H6	-17.448	128.547	
	Purnululu	RMP031	Brain	RNA	H55	-17.448	128.547	
	Purnululu	RMP032	Brain	RNA	H56	-17.448	128.547	
	Purnululu	RMP036	Brain	RNA	H6	-17.448	128.547	
	Purnululu	RMP037	Brain	RNA	H56	-17.448	128.547	
	Purnululu	RMP038	Brain	RNA	H21	-17.448	128.547	
	Purnululu	RMP039	Brain	RNA	H16	-17.448	128.547	
	Purnululu	RMP040	Brain	RNA	H57	-17.448	128.547	
	Purnululu	RMP042	Brain	RNA	H6	-17.448	128.547	
	Purnululu	RMP045	Brain	RNA	H58	-17.448	128.547	
	Purnululu	RMP046	Brain	RNA	H6	-17.448	128.547	
Northern Territory								
	Cape Crawford	RM517	Spleen	RNA	H6	-16.667	135.800	
	Cape Crawford	RM521	Brain	RNA	H21	-16.667	135.800	
	Cape Crawford	RM523	Spleen	RNA	H6	-16.667	135.800	
	Cape Crawford	RM528	Spleen	RNA	H48	-16.667	135.800	
	Cape Crawford	RM539	Brain	RNA	H6	-16.667	135.800	
	Cape Crawford	RM542	Spleen	RNA	H6	-16.667	135.800	
	Cape Crawford	RM544	Brain	RNA	H49	-16.667	135.800	
	Cape Crawford	RM548	Brain	RNA	H6	-16.667	135.800	
	Mataranka	RM510	Brain	RNA	H6	-14.923	133.066	
	Mataranka	RM511	Brain	RNA	H47	-14.923	133.066	This study
	Mataranka	RM515	Brain	RNA	H6	-14.923	133.066	
	Mataranka	RM581	Brain	RNA	H21	-14.923	133.066	
	Timber Creek	RM604	Spleen	RNA	H21	-15.643	130.467	
	Timber Creek	RM607	Spleen	RNA	H21	-15.643	130.467	
	Timber Creek	RM608	Spleen	RNA	H21	-15.643	130.467	
	Timber Creek	RM704	Spleen	RNA	H6	-15.643	130.467	
	Timber Creek	RM712	Brain	RNA	H37	-15.643	130.467	
	Timber Creek	RM713	Brain	RNA	H16	-15.643	130.467	
	Timber Creek	RM714	Brain	RNA	H6	-15.643	130.467	
	Timber Creek	RM726	Brain	RNA	H6	-15.643	130.467	
	Borroloola							Slade and Moritz et al.
Queensland								
	Burketown	RM349	Brain	RNA	H6	-17.852	139.633	
	Burketown	RM350	Brain	RNA	H6	-17.852	139.633	
	Burketown	RM352	Brain	RNA	H39	-17.852	139.633	

	Burketown	RM354	Brain	RNA	H40	-17.852	139.633	
	Cairns	RMP001	Brain	RNA	H43	-16.919	145.778	
	Cairns	RMP003	Brain	RNA	H6	-16.919	145.778	
	Cairns	RMP004	Brain	RNA	H6	-16.919	145.778	
	Cairns	RMP005	Brain	RNA	H6	-16.919	145.778	
	Cairns	RMP006	Brain	RNA	H37	-16.919	145.778	
	Cairns	RMP007	Brain	RNA	H44	-16.919	145.778	
	Cairns	RMP009	Brain	RNA	H6	-16.919	145.778	
	Cairns	RMP010	Brain	RNA	H6	-16.919	145.778	
	Cairns	RMP012	Brain	RNA	H37	-16.919	145.778	
	Cairns	RMP015	Brain	RNA	H45	-16.919	145.778	
	Cairns	RMP016	Brain	RNA	H37	-16.919	145.778	
	Cairns	RMP020	Brain	RNA	H46	-16.919	145.778	
	Croydon	RM299	Brain	RNA	H37	-18.206	142.240	
	Croydon	RM300	Brain	RNA	H6	-18.206	142.240	
	Croydon	RM302	Brain	RNA	H6	-18.206	142.240	
	Croydon	RM304	Brain	RNA	H38	-18.206	142.240	
	Daintree	RM399	Brain	RNA	H41	-16.250	145.317	This study
	Daintree	RM400	Brain	RNA	H6	-16.250	145.317	
	Daintree	RM401	Spleen	RNA	H29	-16.250	145.317	
	Daintree	RM402	Brain	RNA	H42	-16.250	145.317	
	Daintree	RM403	Brain	RNA	H37	-16.250	145.317	
	Daintree	RM404	Spleen	RNA	H37	-16.250	145.317	
	Gordonvale	RM260	Brain	RNA	H6	-17.083	145.796	
	Gordonvale	RM261	Brain	RNA	H34	-17.083	145.796	
	Gordonvale	RM262	Brain	RNA	H35	-17.083	145.796	
	Gordonvale	RM265	Spleen	WGS	H6	-17.083	145.796	
	Gordonvale	RM278	Spleen	RNA	H36	-17.083	145.796	
	Gordonvale	RM280	Brain	RNA	H21	-17.083	145.796	
	Innisfail	RM106	Muscle	RNA	H26	-17.496	146.047	
	Innisfail	RM108	Muscle	RNA	H27	-17.496	146.047	
	Innisfail	RM118	Muscle	RNA, WGS	H6	-17.496	146.047	
	Innisfail	RM127	Muscle	RNA, WGS	H28	-17.496	146.047	
	Innisfail	RM135	Muscle	RNA	H27	-17.496	146.047	
	Rossville	RM169	Muscle	RNA	H29	-15.705	145.223	
	Rossville	RM170	Muscle	RNA	H30	-15.705	145.223	
	Rossville	RM171	Muscle	RNA	H31	-15.705	145.223	
	Rossville	RM179	Muscle	RNA	H32	-15.705	145.223	
	Rossville	RM189	Muscle	RNA	H33	-15.705	145.223	
	Mossman							
	Rockhampton							Slade and Moritz et al.
	Brisbane							
New South Wales								
	Lennox Head							Slade and Moritz et al.
**East of Andes**		KP704699 - KP704700						
		KP979778						
		KP979779 - KP979781						
		KP704701 - KP704713						
		KP704714 - KP704723						
		KP704724						
		KP704725 - KP704734						Acevedo et al.
**West of Andes**		KP704669 - KP704677						
		KP704678 - KP704684						
		KP704685 - KP704691						
		KP704692 - KP704693						
		KP704694 - KP704696						
		KP704697 - KP704698						

Table S2: Mitogenome annotation of *Rhinella marina* with reference genome and the number of parsimony informative sites and singleton sites within 132 individuals.

Gene	Start	Stop	Strand (+/-)	Length	Parsimony informative site	Singleton variable site
tRNA(Leu)	1	72	+	72	0	0
tRNA(Thr)	73	144	+	72	0	0
tRNA(Pro)	144	212	-	69	0	0
tRNA(Phe)	212	279	+	68	0	0
12S ribosomal RNA	280	1214	+	935	5	3
tRNA(Val)	1212	1280	+	69	0	0
16S ribosomal RNA	1281	2885	+	1605	13	6
tRNA(Leu)	2885	2957	+	73	0	0
ND1	2973	3911	+	939	18	6
tRNA(Ile)	3919	3989	+	71	0	0
tRNA(Gln)	3989	4059	-	71	0	0
tRNA(Met)	4059	4127	+	69	0	0
ND2	4128	5156	+	1029	11	4
tRNA(Trp)	5161	5230	+	70	0	0
tRNA(Ala)	5231	5299	-	69	1	0
tRNA(Asn)	5300	5372	-	73	0	0
tRNA(Cys)	5401	5464	-	64	2	0
tRNA(Tyr)	5465	5534	-	70	0	0
COX1	5542	7068	+	1527	20	4
tRNA(Ser)	7082	7152	-	71	0	0
tRNA(Asp)	7154	7222	+	69	1	0
COX2	7224	7895	+	672	5	0
tRNA(Lys)	7912	7983	+	72	1	0
ATP8	7985	8143	+	159	2	2
ATP6	8140	8817	+	678	10	4
COX3	8823	9605	+	783	7	5
tRNA(Gly)	9607	9676	+	70	0	0
ND3	9674	10015	+	342	7	3
tRNA(Arg)	10017	10085	+	69	1	0
ND4L	10125	10382	+	258	4	0
ND4	10379	11737	+	1359	16	4
tRNA(His)	11744	11812	+	69	0	0
tRNA(Ser)	11813	11879	+	67	1	0
ND5	11933	13705	+	1773	20	7
ND6	13707	14198	-	492	5	0
tRNA(Glu)	14199	14266	-	68	0	0
CYTB	14271	15401	+	1131	20	4
Control region	15402	18152		2751	27	11
Intergenic					1	1
				**Total**	**198**	**64**

Table S3: Comparison between WGS and RNASeq-derived mitogenomes.

Sample	Position	Region	WGS	RNASeq	Conversion
RMF048	267	tRNA(F)	A	G	Transition
	2244	23S rRNA	A	T	Transversion
	15524	control region	C	T	Transversion
	15705		T	C	Transition
	15752		C	T	Transition
	15929		C	T	Transition
	15933		T	C	Transition
	15975		T	C	Transition
	16011		A	G	Transition
	16115		A	G	Transition
	16211		A	G	Transition
	16775		G	A	Transition
	16916		T	C	Transition
	17226		C	T	Transition
RMH006	2244	23S rRNA	A	T	Transversion
RMH051	2244	23S rRNA	A	T	Transversion
RMH052	2244	23S rRNA	A	T	Transversion
RM0010	477	16S rRNA	C	T	Transition
	2244	23S rRNA	A	T	Transversion
	5162	tRNA(W)	G	A	Transition
	7162	tRNA(D)	A	T	Transversion
	7920	tRNA(K)	A	T	Transversion
RM0118	2244	23S rRNA	A	T	Transversion
	7162	tRNA(D)	A	G	Transition
	7920	tRNA(K)	A	G	Transition
RM0127	2244	23S rRNA	A	T	Transversion
	5162	tRNA(W)	G	A	Transition
	7162	tRNA(D)	A	T	Transversion
	7920	tRNA(K)	A	G	Transition
RM0045	2244	23S rRNA	A	T	Transversion
	5162	tRNA(W)	G	A	Transition
	7162	tRNA(D)	A	G	Transition
	7920	tRNA(K)	A	G	Transition

Table S4: NUMTs blocks in the cane toad nuclear genome with respect to its mtDNA.

Contig Name	Contig Start	Contig End	Strand	mtDNA Fragment	Fragment Length	Matches	E Value
ctg10165_RHIMB__RM170330.10165	170828	170875	-	17943-17990	48	42	5.00E-05
ctg104_RHIMB__RM170330.104	134299	134336	-	17943-17980	38	36	5.00E-05
ctg11815_RHIMB__RM170330.11815	194991	195051	-	17929-17986	61	52	1.00E-05
ctg1194_RHIMB__RM170330.1194	18743	18780	+	17943-17980	38	36	5.00E-05
ctg1208_RHIMB__RM170330.1208	498924	498989	-	17928-17990	66	55	3.00E-07
ctg1221_RHIMB__RM170330.1221	675608	675653	-	17923-17968	46	41	5.00E-05
ctg12503_RHIMB__RM170330.12503	78151	78290	-	17944-18084	140	107	1.00E-05
ctg12723_RHIMB__RM170330.12723	19348	19385	+	17931-17968	38	36	5.00E-05
ctg1367_RHIMB__RM170330.1367	430982	431026	+	17933-17978	45	42	1.00E-05
ctg13991_RHIMB__RM170330.13991	218571	218608	+	17943-17980	38	36	5.00E-05
ctg15571_RHIMB__RM170330.15571	25520	25559	-	17943-17981	40	38	5.00E-05
ctg16362_RHIMB__RM170330.16362	46563	46600	+	17931-17968	38	36	5.00E-05
ctg16795_RHIMB__RM170330.16795	20291	20334	+	17936-17980	44	41	5.00E-05
ctg18469_RHIMB__RM170330.18469	54795	54837	-	17932-17974	43	39	5.00E-05
ctg19869_RHIMB__RM170330.19869	28837	28882	+	17943-17988	46	41	5.00E-05
ctg23132_RHIMB__RM170330.23132	89407	89455	-	17943-17988	49	45	5.00E-05
ctg24175_RHIMB__RM170330.24175	26491	26532	+	17939-17979	42	40	4.00E-06
ctg24322_RHIMB__RM170330.24322	38880	38929	+	17943-17990	50	45	5.00E-05
ctg2588_RHIMB__RM170330.2588	91193	91240	+	17943-17990	48	42	5.00E-05
ctg2649_RHIMB__RM170330.2649	238602	238662	+	18024-18087	61	53	5.00E-05
ctg26654_RHIMB__RM170330.26654	578	622	-	17933-17980	45	43	1.00E-05
ctg26_RHIMB__RM170330.26	291650	291714	+	17944-18005	65	53	1.00E-05
ctg29742_RHIMB__RM170330.29742	34189	34226	+	17943-17980	38	36	5.00E-05
ctg30050_RHIMB__RM170330.30050	4952	4995	-	17939-17979	44	40	5.00E-05
ctg31312_RHIMB__RM170330.31312	16554	16604	-	17941-17990	51	45	1.00E-05
ctg3490_RHIMB__RM170330.3490	114481	114529	-	17944-17989	49	43	5.00E-05
ctg35_RHIMB__RM170330.35	2052238	2052282	+	17944-17987	45	41	5.00E-05
ctg3738_RHIMB__RM170330.3738	186479	186519	+	17943-17983	41	38	5.00E-05
ctg3992_RHIMB__RM170330.3992	102790	102860	+	17929-17994	71	58	5.00E-05
ctg427_RHIMB__RM170330.427	45564	45608	-	17943-17985	45	41	5.00E-05
ctg4517_RHIMB__RM170330.4517	64852	64896	-	17930-17976	45	42	5.00E-05
ctg4787_RHIMB__RM170330.4787	26493	26542	+	17931-17979	50	44	5.00E-05
ctg5197_RHIMB__RM170330.5197	126698	126745	+	17943-17989	48	43	5.00E-05
ctg5682_RHIMB__RM170330.5682	124532	124576	-	17936-17979	45	41	5.00E-05
ctg626_RHIMB__RM170330.626	23009	23057	-	17943-17990	49	44	1.00E-05
ctg6533_RHIMB__RM170330.6533	64253	64301	+	17942-17991	49	44	5.00E-05
ctg6731_RHIMB__RM170330.6731	62271	62318	+	17943-17990	48	42	5.00E-05
ctg7238_RHIMB__RM170330.7238	69867	69912	+	17943-17987	46	42	1.00E-05
ctg8177_RHIMB__RM170330.8177	10312	10370	+	17931-17988	59	50	1.00E-05
ctg8271_RHIMB__RM170330.8271	35978	36037	-	18028-18087	60	50	4.00E-06
ctg8379_RHIMB__RM170330.8379	8554	8601	+	17943-17988	48	43	1.00E-05
ctg8953_RHIMB__RM170330.8953	85556	85601	-	17943-17988	46	41	5.00E-05
